# A global reptile assessment highlights shared conservation needs of tetrapods

**DOI:** 10.1038/s41586-022-04664-7

**Published:** 2022-04-27

**Authors:** Neil Cox, Bruce E. Young, Philip Bowles, Miguel Fernandez, Julie Marin, Giovanni Rapacciuolo, Monika Böhm, Thomas M. Brooks, S. Blair Hedges, Craig Hilton-Taylor, Michael Hoffmann, Richard K. B. Jenkins, Marcelo F. Tognelli, Graham J. Alexander, Allen Allison, Natalia B. Ananjeva, Mark Auliya, Luciano Javier Avila, David G. Chapple, Diego F. Cisneros-Heredia, Harold G. Cogger, Guarino R. Colli, Anslem de Silva, Carla C. Eisemberg, Johannes Els, Ansel Fong G., Tandora D. Grant, Rodney A. Hitchmough, Djoko T. Iskandar, Noriko Kidera, Marcio Martins, Shai Meiri, Nicola J. Mitchell, Sanjay Molur, Cristiano de C. Nogueira, Juan Carlos Ortiz, Johannes Penner, Anders G. J. Rhodin, Gilson A. Rivas, Mark-Oliver Rödel, Uri Roll, Kate L. Sanders, Georgina Santos-Barrera, Glenn M. Shea, Stephen Spawls, Bryan L. Stuart, Krystal A. Tolley, Jean-François Trape, Marcela A. Vidal, Philipp Wagner, Bryan P. Wallace, Yan Xie

**Affiliations:** 1 Biodiversity Assessment Unit, IUCN-Conservation International, Washington, DC USA; 2 NatureServe, Arlington, VA USA; 3 Smithsonian-Mason School of Conservation and Department of Environmental Science and Policy, George Mason University, Fairfax, VA USA; 4 Instituto de Ecología, Universidad Mayor de San Andrés, La Paz, Bolivia; 5 Université Sorbonne Paris Nord, INSERM, IAME, Bobigny, France; 6 Institute for Biodiversity Science and Sustainability, California Academy of Sciences, San Francisco, CA USA; 7 Institute of Zoology, Zoological Society of London, London, UK; 8 IUCN, Gland, Switzerland; 9 World Agroforestry Center (ICRAF), University of The Philippines, Los Baños, The Philippines; 10 Institute for Marine & Antarctic Studies, University of Tasmania, Hobart, Tasmania Australia; 11 Center for Biodiversity, Temple University, Philadelphia, PA USA; 12 Science & Data Centre: Biodiversity Assessment & Knowledge Team, IUCN, Cambridge, UK; 13 Conservation and Policy, Zoological Society of London, London, UK; 14 Animal, Plant and Environmental Sciences, University of the Witwatersrand, Johannesburg, South Africa; 15 Bishop Museum, Honolulu, HI USA; 16 Department of Herpetology, Zoological Institute, St Petersburg, Russian Federation; 17 Department of Herpetology, Leibniz Institute for the Analysis of Biodiversity Change, Zoological Research Museum Alexander Koenig, Bonn, Germany; 18 Grupo Herpetología Patagónica (GHP-LASIBIBE), Instituto Patagónico para el Estudio de los Ecosistemas Continentales (IPEEC-CONICET), Puerto Madryn, Argentina; 19 School of Biological Sciences, Monash University, Clayton, Victoria Australia; 20 Colegio de Ciencias Biológicas y Ambientales, Museo de Zoología, Instituto de Biodiversidad Tropical iBIOTROP, Universidad San Francisco de Quito USFQ, Quito, Ecuador; 21 Instituto Nacional de Biodiversidad, Quito, Ecuador; 22 Australian Museum Research Institute, Sydney, New South Wales Australia; 23 Departamento de Zoologia, Universidade de Brasília, Brasília, Brazil; 24 South Asia Regional Office, Crocodile Specialist Group, Gampols, Sri Lanka; 25 Charles Darwin University, Darwin, Northern Territory Australia; 26 Environment and Protected Areas Authority, Government of Sharjah, Sharjah, United Arab Emirates; 27 Centro Oriental de Ecosistemas y Biodiversidad (BIOECO), Museo de Historia Natural “Tomás Romay”, Santiago de Cuba, Cuba; 28 Conservation Science & Wildlife Health, San Diego Zoo Wildlife Alliance, San Diego, CA USA; 29 Department of Conservation, Wellington, New Zealand; 30 Institut Teknologi Bandung, Bandung, Indonesia; 31 Department of Biosphere-Geosphere Science, Okayama University of Science, Okayama, Japan; 32 National Institute for Environmental Studies, Tsukuba, Japan; 33 Departamento de Ecologia, Universidade de São Paulo, São Paulo, Brazil; 34 School of Zoology & the Steinhardt Museum of Natural History, Tel Aviv University, Tel Aviv, Israel; 35 School of Biological Sciences, The University of Western Australia, Crawley, Western Australia Australia; 36 Zoo Outreach Organization, Coimbatore, India; 37 Departamento de Zoología, Universidad de Concepción, Concepción, Chile; 38 Chair of Wildlife Ecology and Management, University of Freiburg, Freiburg, Germany; 39 Museum für Naturkunde - Leibniz Institute for Evolution and Biodiversity Science, Berlin, Germany; 40 Chelonian Research Foundation, Arlington, VT USA; 41 Museo de Biología, Universidad del Zulia, Maracaibo, Venezuela; 42 Ben-Gurion University of the Negev, Midreshet Ben-Gurion, Israel; 43 University of Adelaide, Adelaide, South Australia Australia; 44 Facultad de Ciencias, UNAM, Mexico City, Mexico; 45 Sydney School of Veterinary Science B01, University of Sydney, Sydney, New South Wales Australia; 46 Section of Research & Collections, North Carolina Museum of Natural Sciences, Raleigh, NC USA; 47 South African National Biodiversity Institute, Cape Town, South Africa; 48 Institut de Recherche pour le Développement, MIVEGEC, Dakar, Senegal; 49 Departamento de Ciencias Básicas, Facultad de Ciencias, Universidad del Bío-Bío, Chillán, Chile; 50 Allwetterzoo, Münster, Germany; 51 Ecolibrium, Inc., Boulder, CO USA; 52 Chinese Academy of Sciences, Beijing, China

**Keywords:** Conservation biology, Herpetology

## Abstract

Comprehensive assessments of species’ extinction risks have documented the extinction crisis^1^ and underpinned strategies for reducing those risks^2^. Global assessments reveal that, among tetrapods, 40.7% of amphibians, 25.4% of mammals and 13.6% of birds are threatened with extinction^3^. Because global assessments have been lacking, reptiles have been omitted from conservation-prioritization analyses that encompass other tetrapods^4–7^. Reptiles are unusually diverse in arid regions, suggesting that they may have different conservation needs^6^. Here we provide a comprehensive extinction-risk assessment of reptiles and show that at least 1,829 out of 10,196 species (21.1%) are threatened—confirming a previous extrapolation^8^ and representing 15.6 billion years of phylogenetic diversity. Reptiles are threatened by the same major factors that threaten other tetrapods—agriculture, logging, urban development and invasive species—although the threat posed by climate change remains uncertain. Reptiles inhabiting forests, where these threats are strongest, are more threatened than those in arid habitats, contrary to our prediction. Birds, mammals and amphibians are unexpectedly good surrogates for the conservation of reptiles, although threatened reptiles with the smallest ranges tend to be isolated from other threatened tetrapods. Although some reptiles—including most species of crocodiles and turtles—require urgent, targeted action to prevent extinctions, efforts to protect other tetrapods, such as habitat preservation and control of trade and invasive species, will probably also benefit many reptiles.

## Abstract (web summary)

An extinction-risk assessment of reptiles shows that at least 21.1% of species are threatened by factors such as agriculture, logging, urban development and invasive species, and that efforts to protect birds, mammals and amphibians probably also benefit many reptiles.

## Main

Although comprehensive extinction-risk assessments have been available for birds, mammals and amphibians for well over a decade^3^, reptiles have, until now, not been comprehensively assessed. Therefore, conservation science and practice has typically relied on the International Union for Conservation of Nature (IUCN) Red List categories and distributions of the other three tetrapod classes to inform policy and guide priorities for investments^2^, despite differing expectations as to how effective common strategies will be across classes^9,10^. With a high diversity in arid regions and some islands and archipelagos (for example, Antilles, New Caledonia and New Zealand) compared with other tetrapods, reptiles were thought to require different conservation strategies and geographical priorities^6^. In the absence of Red List assessments, researchers have resorted to indirect measures of extinction risk such as range size and human pressure^6,11^. Here we examine the results of a comprehensive Red List assessment of reptiles and outline their implications for the conservation needs of reptiles.

Comprising the turtles (Testudines), crocodiles (Crocodylia), squamates (Squamata: lizards, snakes and amphisbaenians) and tuatara (Rhynchocephalia), reptiles are a paraphyletic class representing diverse body forms, habitat affinities and functional roles in their respective ecosystems^12^. The largely terrestrial squamates are by far the most speciose group (9,820 species in this assessment), whereas the primarily aquatic turtles and crocodiles are often larger bodied but include only 351 and 24 species, respectively. Rhynchocephalians diverged from the snake and lizard lineage in the Triassic period and include one extant species^13^. Given this diversity of reptiles, threats to their persistence are likely to be equally varied, and so these need to be specified to guide effective conservation action.

## Extinction risk and threats

**Fig. 1:**
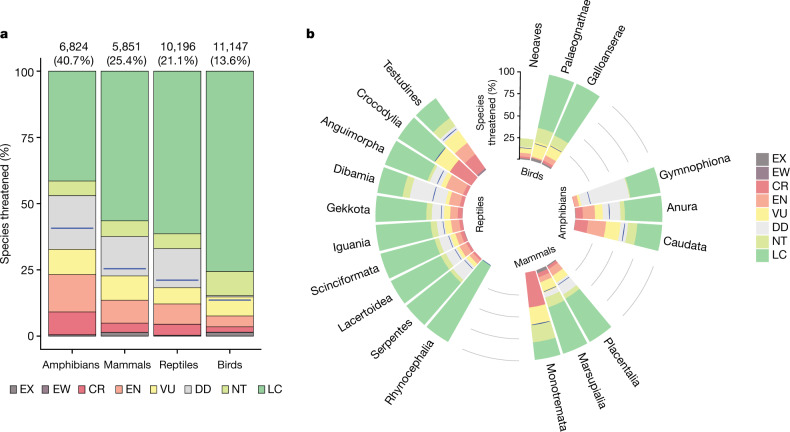
Taxonomic patterns of extinction risk in tetrapods. **a**, Taxonomic patterns organized by class. The numbers above each column refer to the numbers and percentages of species threatened (that is, those categorized as critically endangered, endangered or vulnerable). **b**, Extinction risk by major taxonomic groups. Blue lines indicate the best estimate of the percentage of species threatened. CR, critically endangered; DD, data deficient; EN, endangered; EW, extinct in the wild; EX, extinct; LC, least concern; NT, near threatened; VU, vulnerable. Source data

**Extended Data Fig. 1:**
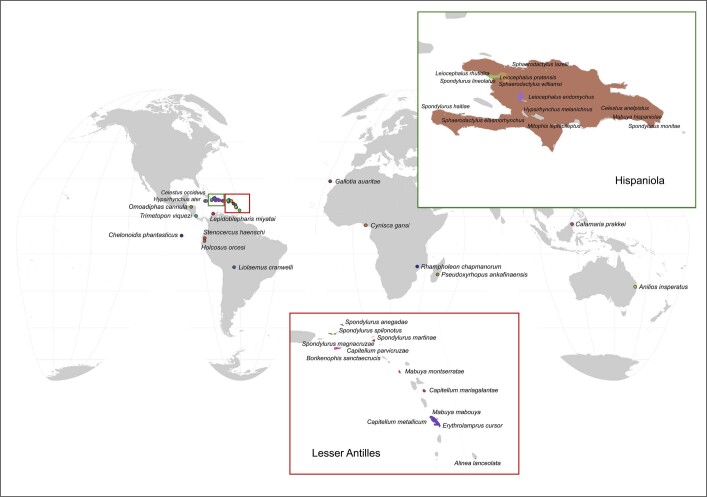
Locations of critically endangered (possibly extinct) reptiles. Colours depict individual species’ ranges. These species were classified as critically endangered (possibly extinct) (CR(PE)) at the time of their assessments. Fortunately, a few species such as *Rhampholeon chapmanorum*^59^ have been recorded subsequent to their assessment publication dates and will no longer be classified as CR(PE) once they are reassessed in the future.

We assessed reptiles globally using the IUCN Red List criteria with input from 961 scientists (Supplementary Note 1) achieved primarily through 48 workshops (Supplementary Table 1). Across all 10,196 species assessed, 21.1% are threatened with extinction (categorized as vulnerable, endangered or critically endangered; Supplementary Table 2). As a group, a greater number of reptile species are threatened than birds or mammals, but fewer than amphibians. Proportionately more mammals and amphibians are threatened than reptiles (Fig. 1a). The reptile threat prevalence falls within a previous estimate of 15–36% threatened (best estimate 19%) from a random sample of 1,500 reptile species^8^. To our knowledge, this study represents the first global test of a sampled Red List extrapolation. The proportion of turtles and crocodiles that are threatened (57.9% and 50.0%, respectively) is much higher than those of squamates (19.6%) and tuatara (0%), and comparable to the most-threatened tetrapod groups, salamanders (57.0%) and monotremes (60.0%) (Fig. 1b). Within squamates, iguanid (73.8%) and xenosaurid (60.0%) lizards and uropeltid (61.1%) and tropidophiid (60.0%) snakes are highly threatened. Since 1500, 31 reptile species (0.3%) have been driven extinct, including 24 squamates and 7 turtles, with 2 squamate species from Christmas Island categorized as extinct in the wild (persisting only as captive populations); 40 critically endangered species are ‘possibly extinct’ (that is, species that are likely to be extinct, but that have a small chance that they may be extant; Extended Data Fig. 1). Additional species probably became extinct before being documented by science^14^.

Limited information resulted in 1,507 species (14.8%) being categorized as data deficient, similar to the number in mammals (15.1%) and lower than for amphibians (20.4%), but much higher than for birds (0.5%). Taxonomic groups with fossorial or other secretive habits and/or restricted to poorly studied areas (such as blindsnakes (Gerrhopilidae and Typhlopidae)) had greater proportions of species categorized as data deficient (Supplementary Table 2). The greatest numbers of data-deficient species occur in Asia (585), South America (284) and Africa (271), with fewer data-deficient species in North and Middle America (163), Oceania (124), Australia (55), the Caribbean (34) and Europe (3). Uncertainty about the status of data-deficient species suggests that the proportion of reptiles threatened with extinction ranges from 18.0% (assuming no data-deficient species are threatened) to 32.8% (assuming all data-deficient species are threatened) with a best estimate of 21.1%.

**Fig. 2:**
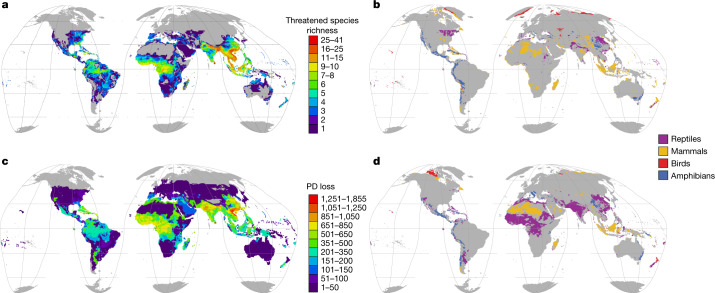
Geographical patterns of threat in reptiles and other tetrapods in terrestrial regions. **a**, Distribution of reptile species that are threatened (critically endangered, endangered or vulnerable). **b**, Regions with disproportionate numbers of threatened species for each tetrapod class (areas for each class where the proportional threat in species diversity is at least twice the loss for the next-most threatened class). **c**, Loss of reptile phylogenetic diversity (PD) if all threatened species became extinct. **d**, Regions with disproportionate phylogenetic diversity loss for each tetrapod class (calculated as in **b**). Grey, areas with no threatened species (**a**, **c**) or regions in which no class is disproportionately threatened (**b**, **d**). Data are shown at a resolution of 50 km.

**Extended Data Fig. 2:**
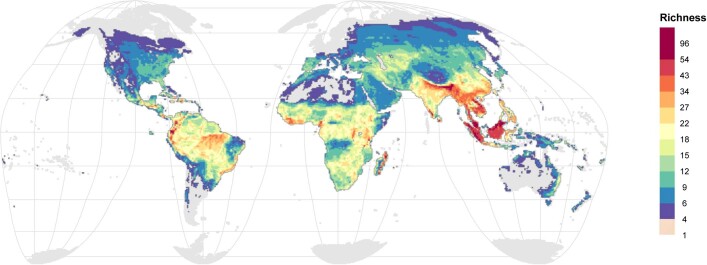
Richness of threatened (CR, EN, VU) tetrapods (50-km resolution). As in Fig. 2a, only threatened species occurring in terrestrial areas are shown.

Concentrations of threatened reptiles are mostly in regions in which other tetrapods are also threatened (Extended Data Fig. 2). Threatened reptiles are concentrated in southeastern Asia, West Africa, northern Madagascar, the northern Andes and the Caribbean, but largely absent from Australian drylands; the Kalahari, Karoo and Sahara deserts; northern Eurasia; and the Rocky Mountains and northern North America (Fig. 2a). In remarkably few regions, however, are reptiles disproportionately threatened relative to other tetrapods (that is, have at least twice the number of species in a threatened category): parts of southern Asia and northeastern USA (Fig. 2b and Extended Data Table 1). Moreover, for most (87%) terrestrial regions in which tetrapods occur, no tetrapod class is disproportionately threatened compared with the other classes.

**Extended Data Fig. 3:**
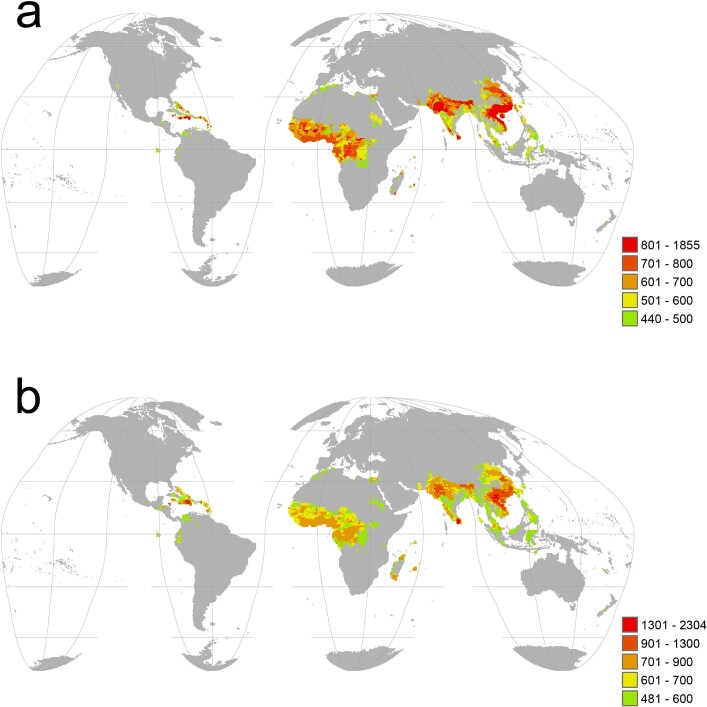
Areas with the top 15% of PD loss if all threatened reptiles become extinct. **a**, 50-km resolution, **b**, 100-km resolution.

With deep phylogenetic lineages and high species diversity, reptiles stand to lose a large amount of phylogenetic diversity (a measure of difference within an evolutionary tree^15^) if the current extinction crisis continues apace. Assuming all threatened species (and only these species) become extinct, the combined loss of reptile phylogenetic diversity (calculated using existing phylogenetic trees^16,17^) will be approximately 15.6 billion years. Southeastern Asia, India, West Africa and the Caribbean^8^ (Fig. 2c) comprise the top 15% areas of phylogenetic diversity loss, with high concentrations of threatened and evolutionarily distinct species (Extended Data Fig. 3). Comparing the distributions of threatened phylogenetic diversity across all four tetrapod groups reveal relatively small geographical areas of disproportionate importance for any class (Fig. 2d and Extended Data Table 2).

**Fig. 3:**
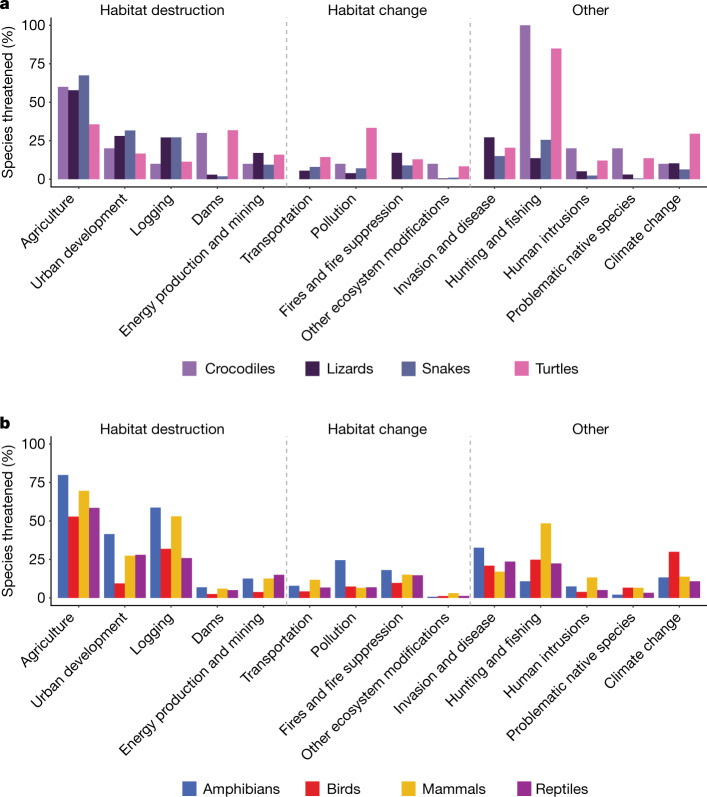
Threats to reptiles and other tetrapods. **a**, Crocodiles, lizards (including amphisbaenians), snakes and turtles. **b**, All tetrapods. Only threats to species categorized as critically endangered, endangered or vulnerable were included. Some species are subject to more than one threat (mean = 2.4; s.d.  = 1.3 threats per species). Source data

The anthropogenic factors increasing extinction risk in reptiles are mainly habitat destruction from agricultural expansion, urban development and logging (Fig. 3a). Other important threats are invasive species and hunting, which includes commercial harvest and trade (Fig. 3a). Among reptile groups, crocodiles and turtles are most frequently affected by hunting and less by agriculture, whereas squamates are most frequently threatened by agriculture (Fig. 3a). The major threats are broadly similar across tetrapods (Fig. 3b). For all tetrapod groups, agriculture threatens the most species, logging is the second or third most prevalent threat, and invasive species and disease are the fourth or fifth most prevalent threat. Threats causing habitat destruction (complete removal of habitat) affect proportionately more species than those causing habitat change (degradation of habitat). The largest differences in relative threat prevalence are for hunting, which threatens mammals much more than the other tetrapods, and urban development, which affects amphibians, reptiles and mammals more than birds.

Climate change is a looming threat to reptiles, for example, by reducing thermally viable windows for foraging^18^, skewing offspring sex ratios in species that have temperature-dependent sex determination^19^ and contracting ranges^20^. Given the Red List three-generation horizon for assessments, the lack of long-term studies limits the documentation of climate change as a near-future threat to reptiles^21^, in contrast to, for example, birds (Fig. 3b). Disease is documented as a threat for only 11 species of reptiles (<1% of extant, non-data-deficient species), although pathogens such as *Ophidiomyces ophiodiicola* (which causes snake fungal disease^22^) pose a potential threat and are little studied outside North America. Intentional use of reptiles (local consumption and trade) is an important threat to reptiles^23^, and was found to threaten 329 species (3.2%), especially turtles (30.8% of all turtle species).

**Fig. 4:**
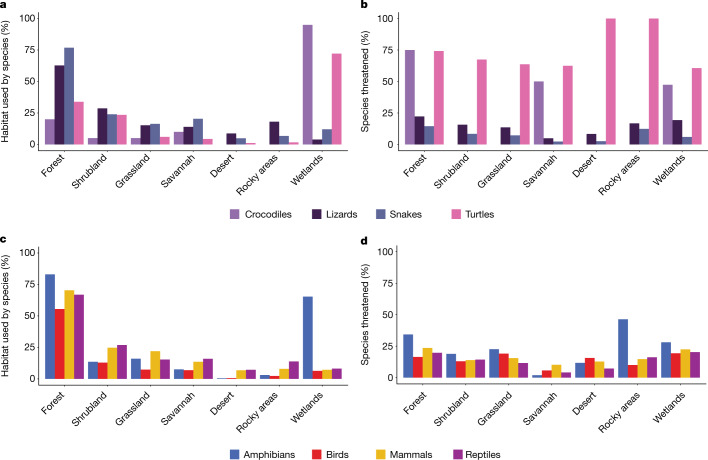
Habitat use by reptiles and other tetrapods. **a**, Habitats used by crocodiles, lizards (includes amphisbaenians), snakes and turtles. **b**, Percentage of reptiles using each habitat that are threatened. **c**, Habitats used by tetrapods. **d**, Percentage of threatened tetrapod species using each habitat. See Supplementary Table 3 for additional, rarely used habitats not shown here. Artificial habitats are not shown. Source data

More than half of all reptile species occur in forested habitats (Fig. 4c). Although some reptiles, particularly lizards, are speciose in arid or seasonally dry habitats such as deserts, grasslands, shrublands and savannahs^6,24^, these species are less threatened than those occupying forest habitats (13.7% of species restricted to arid habitats versus 26.6% of species restricted to forests; Fisher’s exact test, *P* = 0.00001; Fig. 4b). The top threats to reptiles—agriculture, urban development and logging—are also the top threats to species inhabiting forested habitats, affecting 65.9%, 34.8% and 27.9% of forest-dwelling threatened reptiles, respectively, helping to explain the higher extinction risk of forest species. Agriculture and logging are significantly more likely to threaten forest-dwelling than non-forest dwelling reptiles (Fisher’s exact test, *P* = 0.00001), whereas urbanization threatens forest-dwelling no differently than non-forest dwelling reptiles (Fisher’s exact test, *P* = 0.25). Turtles and crocodiles are much more frequently associated with wetlands than other reptiles (Fig. 4a).

Like reptiles, more than twice as many bird and mammal species occur in forests compared with any other habitat type (Fig. 4c). Forests are also the most common habitat for amphibians, although wetlands are important for many species, especially for breeding (Fig. 4c). Also similar to reptiles, the proportions of forest-inhabiting bird, mammal and amphibian species that are threatened are higher than for species that do not inhabit forests (16.7% versus 13.0%, 27.5% versus 20%, and 42.4% versus 34.4%, respectively; Fisher’s exact tests, *P* = 0.00001). Threat levels for each tetrapod class in arid habitats tend to be lower (less than 23% of species occurring in such regions). Across tetrapods, forests support high diversity and are also subject to widespread threats.

## Surrogacy of other tetrapods for reptiles

**Extended Data Fig. 4:**
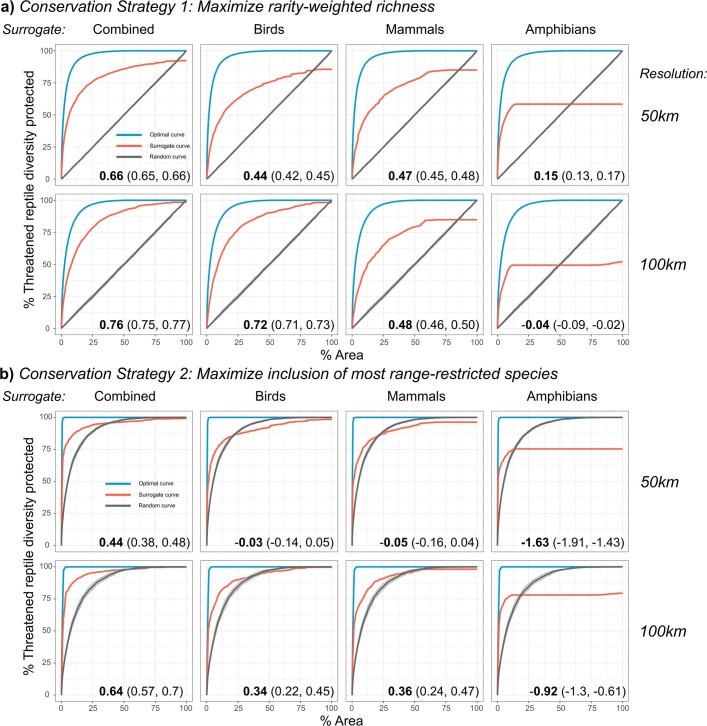
Species Accumulation Index (SAI) values and curves for surrogacy. analysis. Effectiveness of threatened birds, mammals, and amphibians – individually or combined – as a surrogate for the conservation of threatened reptile species diversity for two alternative strategies – **a**, Conservation Strategy 1: Maximize rarity-weighted richness, **b**, Conservation Strategy 2: Maximize inclusion of most range-restricted species. See Methods for full explanation of each strategy. Surrogate effectiveness is measured using the Species Accumulation Index (SAI): values range from −∞ to 1, with 1 indicating perfect surrogacy, values between 1 and 0 indicate positive surrogacy, 0 indicating no surrogacy, and values less than 0 indicating negative surrogacy. In each panel, median SAI values are in bold, with lower and upper confidence intervals in brackets. Blue lines are the optimal curves (accumulation of target diversity based on target priority areas); red lines are the surrogate curves (accumulation of target diversity based on surrogate priority areas); and grey lines are the random curves (accumulation of target diversity based on random selection of areas). Confidence intervals (95%, based on 100 randomizations) shown in lighter shading around curves; most are too small to be visible.

With numerous threatened tetrapod species (227 birds, 194 mammals, 607 amphibians and 474 reptiles) ranging completely outside formally protected areas, assessing surrogacy is important to gauge the magnitude of efforts needed to conserve these species. We addressed surrogacy using a complementarity representation approach for threatened species, which better addresses the extent to which areas selected for surrogates capture target features than, for example, spatial congruence^25^. When combined, threatened birds, mammals and amphibians—the tetrapod groups for which comprehensive Red List data were previously available—are good surrogates for the conservation of threatened reptile diversity when prioritizing the richness of rarity-weighted threatened species at both 50-km and 100-km resolution (median species accumulation indices are 0.66 and 0.76, respectively; Extended Data Fig. 4a). Using this same prioritization strategy, birds and mammals individually are reasonable surrogates for reptiles, whereas amphibians are poor surrogates (Extended Data Fig. 4a). By contrast, for a complementarity representation strategy that prioritizes individual threatened species with the smallest ranges, birds, mammals and amphibians are not good surrogates for reptiles (species accumulation indices < 0.40), although combined they are reasonable surrogates at both 50-km and 100-km resolutions (median species accumulation indices are 0.44 and 0.64, respectively; Extended Data Fig. 4b). These results indicate that the smallest-ranged threatened reptiles tend to be isolated from threatened birds, mammals and amphibians. In addition, priority areas for threatened birds and mammals independently, and birds, mammals and amphibians combined, showed high spatial congruence with priority sites for threatened reptiles for both strategies (prioritizing either rarity-weighted threatened species or complementarity representation), although correlations among global portfolios of priority areas were lower (Extended Data Tables 3, 4). Although our results for the smallest ranged threatened species are consistent with previous expectations of low surrogacy^9^, overall, we found reasonably high surrogacy^10^.

## Discussion

Our discovery of broad similarities in the geography and nature of threats between reptiles and other tetrapods was unexpected given previous arguments about the exceptionalism of reptiles for being particularly diverse in arid habitats^6^. The implications for tetrapod conservation are that geographical prioritizations previously performed for birds, mammals and amphibians overlap broadly with prioritizations for all except the most range-restricted threatened reptiles. The absence of reptiles in many global conservation prioritization analyses to date is unlikely to have left the class less represented than others. Nevertheless, the low surrogacy value of other tetrapods for reptiles with the most restricted ranges suggests that a case-by-case focus is required for these microendemics. Indeed, the ranges of 31 threatened reptiles do not overlap with the ranges of any other threatened tetrapod (among threatened species, 84 birds, 11 mammals and 7 amphibians are similarly isolated from other threatened tetrapods) (Supplementary Table 3).

Researchers have predicted that reptiles are particularly vulnerable to climate change in tropical biomes^26^ as well as freshwater^27^ and arid habitats^18^, although so far no clear geographical signal in reptile declines due to climate change has been detected^28^. If such vulnerabilities are found, then—as climate change continues to alter the distributions and extinction risk of species—the surrogacy across tetrapods could unravel with, for example, reptiles in specific habitat types declining swiftly and disproportionately (relative to other tetrapods).

Among the conservation strategies needed to prevent reptile extinction, land protection is critically important to buffer many threatened species from the dual threats of agricultural activities and urban development. The hundreds of threatened reptiles that currently occur completely outside protected areas underscore the need for targeted safeguards of important sites. Beyond place-based strategies, conservation policy and practice must halt unsustainable harvest and stem the spread of invasive disease to prevent many more species from becoming threatened^23^. Furthermore, introduced mammals to islands threaten 257 reptile species (2.8% of all reptiles), calling for continued campaigns to eradicate introduced mammals in those places.

With a comprehensive, global assessment of the extinction risk of reptile species now available, these data can be incorporated into the toolbox of conservation practice and policy. At the species level, they can serve as the starting point for ‘green status’ (formerly ‘green list’) assessments that define, measure and incentivize species recovery^29^. More generally, they can be integrated into the calculation of species threat abatement and restoration metrics^2^, the identification of key biodiversity areas^30^ and resource allocation using systematic conservation planning^31^, all of which have primarily been dependent on data from birds, mammals and amphibians among animals to date. Future reassessments will allow reptile data to be included in the Red List Index^32^, a widely used indicator of biodiversity trends^1^.

Although efforts aimed at protecting other threatened tetrapods probably benefit many of the 1,829 threatened reptiles—especially forest-dwelling species—conservation investments targeted at uniquely occurring reptiles or those requiring tailored policies must also be implemented to prevent extinction. Encouragingly, the First Draft of the Post-2020 Global Biodiversity Framework^33^ to be agreed by governments in 2022 explicitly targets safeguarding important sites (target 3), complemented by emergency action for individual threatened species (target 4). This political determination to reverse the slide of species toward extinction bodes well for reptiles.

## Methods

We used the IUCN Red List criteria^34,35^ and methods developed in other global status-assessment efforts^36,37^ to assess 10,078 reptile species for extinction risk. We additionally include recommended Red List categories for 118 turtle species^38^, for a total of 10,196 species covered, representing 89% of the 11,341 described reptile species as of August 2020^39^.

### Data compilation

We compiled assessment data primarily through regional in-person and remote (that is, through phone and email) workshops with species experts (9,536 species) and consultation with IUCN Species Survival Commission Specialist Groups and stand-alone Red List Authorities (442 species, primarily marine turtles, terrestrial and freshwater turtles, iguanas, sea snakes, mainland African chameleons and crocodiles). We conducted 48 workshops between 2004 and 2019 (Supplementary Table 1). Workshop participants provided information to complete the required species assessment fields (geographical distribution, population abundance and trends, habitat and ecological requirements, threats, use and trade, literature) and draw a distribution map. We then applied the Red List criteria^34^ to this information to assign a Red List category: extinct, extinct in the wild, critically endangered, endangered, vulnerable, near threatened, least concern and data deficient. Threatened species are those categorized as critically endangered, endangered and vulnerable.

### Taxonomy

We used The Reptile Database^39^ as a taxonomic standard, diverging only to follow well-justified taxonomic standards from the IUCN Species Survival Commission^40^. We could not revisit new descriptions for most regions after the end of the original assessment, so the final species list is not fully consistent with any single release of The Reptile Database.

### Distribution maps

Where data allowed, we developed distribution maps in Esri shapefile format using the IUCN mapping guidelines^41^ (1,003 species). These maps are typically broad polygons that encompass all known localities, with provisions made to show obvious discontinuity in areas of unsuitable habitat. Each polygon is coded according to species’ presence (extant, possibly extant or extinct) and origin (native, introduced or reintroduced)^41^. For some regions covered in workshops (Caucasus, Southeast Asia, much of Africa, Australia and western South America), we collaborated with the Global Assessment of Reptile Distributions (GARD) (http://www.gardinitiative.org/) to provide contributing experts with a baseline species distribution map for review. Although refined maps were returned to the GARD team, not all of these maps have been incorporated into the GARD.

### Habitat preferences

Where known, species habitats were coded using the IUCN Habitat Classification Scheme (v.3.1) (https://www.iucnredlist.org/resources/habitat-classification-scheme). Species were assigned to all habitat classes in which they are known to occur. Where possible, habitat suitability (suitable, marginal or unknown) and major importance (yes or no) was recorded. Habitat data were available for 9,484 reptile species.

### Threats

All known historical, current and projected (within 10 years or 3 generations, whichever is the longest; generation time estimated, when not available, from related species for which it is known; generation time recorded for 76.3% of the 186 species categorized as threatened under Red List criteria A and C1, the only criteria using generation length) threats were coded using the IUCN Threats Classification Scheme v.3.2 (https://www.iucnredlist.org/resources/threat-classification-scheme), which follows a previously published study^42^. Where possible, the scope (whole (>90%), majority (50–90%), minority (<50%) of the population; unknown) and severity (causing very rapid (>30%), rapid (>20%), slow but notable (<20%) declines over 10 years or 3 generations, whichever is longer; negligible declines; unknown) of the threat was recorded. Threat data were available for 1,756 of the 1,829 threatened reptile species.

### Assessment review

Each assessment underwent two reviews. First, a scientist familiar with the species but not involved in the assessment reviewed the account for biological accuracy and accurate application of the Red List criteria. Once the assessors revised the assessment satisfactorily, staff from the IUCN Red List Unit reviewed the assessment primarily for accurate application of the Red List criteria. The assessors revised the assessment again, if necessary, to satisfy any concerns of the IUCN Red List Unit before the assessment was finalized.

### Data limitations

Although we made an extensive effort to complete assessments for all reptiles, some data gaps remain.

#### Missing species

As of December 2020, 1,145 reptile species, primarily snakes and lizards, were omitted from the present study, including the phylogenetic diversity analyses, because they were described recently and they were described after previous comprehensive assessments from the region. Geographically, they are primarily from tropical regions (as are assessed reptiles) with an underrepresentation of African species (distribution of omitted species: Asia, 41%; Africa, 8%; Australia, 7%; Europe, 3%; North/Central America, 20%; South America, 19%; Caribbean, 5%; Oceania, 4%; percentages sum to greater than 100% because some species occur in two regions). Because they are recently described, many are poorly known, may be rare or occur in a very restricted area, or in poorly surveyed areas that are often subject to high levels of human impacts. As such, recent descriptions are more likely to receive a data-deficient or threatened Red List category than be assigned of least concern^41^. The net effect on our analyses is a slight underestimate of the number of threatened snakes and lizards, and plausibly a slight overestimate of least concern species. With tetrapod species described in the future likely to be small-ranged, threatened lizards and amphibians^43,44^, surrogacy levels may decline from those reported here.

#### Geographical coverage

Although we made extensive efforts to map the current known distribution for each species, this information is incomplete for some species. Where appropriate, and following expert guidance, we interpolated between known localities if the ecological conditions appeared appropriate. In addition, species occurrence is unlikely to be spread evenly or entirely throughout the area depicted in range maps, with gaps expected, for example, in patches of unsuitable habitat.

#### Data-deficient species

For species assessed to be data deficient (1,507 reptiles, 14.8%), there was inadequate information on the distribution, population status or threats (historical, current or projected future) of the species (both from published sources and expert knowledge) to make a direct, or indirect, assessment of the risk of extinction. All species were assessed according to their recognized taxonomic circumscription at the time of assessment. Taxonomic uncertainty therefore did not result in a data-deficient assignment, although some species were listed as data deficient because they are morphologically indistinguishable from another species and therefore estimates of distribution and abundance are not feasible.

#### Time span of assessments

The assessments were completed between 1996 and 2020, with 1,503 assessments completed before 2011. The IUCN Rules of Procedure (https://www.iucnredlist.org/resources/rules-of-procedure) recommend reassessment every 10 years and thus, as of 2020, 15% of the assessments can be considered outdated. Of the species assessed 1996–2010, slightly more species were threatened (23.0%) than the species assessed more recently (20.7%). This difference is largely explained by the greater percentages of crocodiles and turtles with outdated assessments (29% and 35%, respectively) compared with tuatara, lizards and snakes (0%, 12% and 17%, respectively) and the highly threatened nature of crocodiles and turtles (Supplementary Table 2). The continuing deterioration of biodiversity globally^1^ suggests that the species with outdated assessments are more likely to be in higher threat categories today than when they were when last assessed, causing an underestimation of current reptile threat status.

### Analyses

#### Percentage of species threatened with extinction

To estimate the percentage of species threatened with extinction (categories critically endangered, endangered and vulnerable), we used the following formula, which assumes that data-deficient species have the same proportion of threatened species as species that were not data deficient.

$${{\rm{Prop}}}_{{\rm{threat}}}=\frac{{\rm{CR}}+{\rm{EN}}+{\rm{VU}}}{N-{\rm{DD}}}$$

where Prop_threat_ is the best estimate of the proportion of species that are threatened; CR, EN, VU and DD are the number of species in each corresponding Red List category and *N* is the number of species assessed (excluding extinct and extinct in the wild species).

#### Data for amphibians, birds and mammals

For all analyses that included data for amphibians, birds and mammals, we used the 2020-1 version^3^ of the tabular and spatial data downloaded from the IUCN Red List website in May 2020.

#### Threats

Threats calculations were restricted to species in threatened Red List categories (critically endangered, endangered and vulnerable). Multiple threats can affect a single species. Summaries of threats are for the first level of the IUCN classification scheme. Threats thought to affect only a minority of the global population (<50% of the population) (coded as ‘minority’) were not included. In addition, we removed threats that were assessed to cause ‘no declines’ and ‘negligible declines’ from the analysis (as indicated by the severity coding). We considered all threats without scope or severity scored to be major threats and retained them in the analysis.

#### Habitat

Analyses of habitat use were restricted to the first level of the IUCN habitat-classification scheme. We excluded habitats for which the major importance to the species was scored ‘no’ and suitability was scored ‘marginal’ and considered all habitats without major importance or suitability scored to be suitable and of major importance and included them in the analyses. We did not consider artificial habitats in the analyses.

Only a small number of reptile species inhabits ‘caves/subterranean’ and ‘marine coastal’ habitats, so they were not included in Fig. 4 but their threat prevalence is summarized in Supplementary Table 4.

#### Statistics

Statistical tests were designed to avoid inclusion of multiple observations from the same species (because species can occur in multiple habitats and be threatened by multiple threats). To assess whether arid habitat or forest species were more likely to be threatened, we included only species that were restricted to one of these habitat types. For threats analyses, we compared species that occur in forests (including those that occur in forests and other habitats) to those that do not occur in forests. All tests were two-tailed Fisher’s exact tests.

#### Geographical patterns

The geographical patterns of threat and phylogenetic diversity shown in Fig. 2 are for only terrestrial species (so, for reptiles, excluding 87 species of marine turtles and sea snakes). Tetrapod classes vary widely in the numbers of pelagic marine species and in the methods used to map distributions. Restricting analyses to terrestrial species ensured more-consistent analyses and avoided wide variation in summary values caused by small numbers of species.

Analyses of the distribution maps used polygons either with the following IUCN map code designations or with no codes indicated:

Presence = extant (code 1) and probably extant (code 2)

Origin = native (code 1), reintroduced (code 2) and introduced (code 6)

Seasonality = resident (code 1), breeding season (code 2), non-breeding season (code 3) and passage (code 4).

Ranges for species categorized as critically endangered (possibly extinct) are coded as possibly extinct (code 4) and excluded from the spatial analyses.

All spatial analyses were conducted on a global 0.5° by 0.5 ° latitude–longitude grid (approximately 50 km at the Equator). To explore the influence of spatial resolution, we repeated the surrogacy and phylogenetic diversity analyses at a 100-km resolution. We converted polygon range maps (tagged with the appropriate codes as described above) to these grids. We used a global equal-area pseudocylindrical projection, Goode homolosine.

We mapped the distribution of threatened species as a count of the number of species with ranges overlapping each grid cell.

#### Estimating the spatial distribution of disproportionate threat and phylogenetic diversity loss

We identified global areas in which each tetrapod class is disproportionately threatened compared with all other classes by comparing the species-richness-adjusted level of threat among the four tetrapod classes. First, for each grid cell, we identified the proportional threat level of each class by dividing the number of species in threatened Red List categories (vulnerable, endangered and critically endangered) by the total number of species for the class found in that cell. Second, for all grid cells in which at least five tetrapod species are present, we compared proportional threat values across the four classes and identified a grid cell as having a disproportionate threat level for a given class if: (1) the grid cell had a proportional level of threat equal to 10% or higher for the class; and (2) the grid cell had a proportional level of threat for the class at least twice as high as the proportional level of threat for the next class. We assessed the sensitivity of disproportionate threat patterns to our definition of disproportionate threat by varying the degree of difference in proportional threat level between the highest and second highest class. We identified the number of grid cells with disproportionate threat for each class when the class had a proportional threat level: (1) higher than any other class; (2) 25% or more higher than any other class; (3) 50% or more higher than any other class; (4) 100% or more higher than any other class; and (5) 200% or more higher than any other class. In the main text, we report results for the 100% or more threat level. Results for all thresholds are included in Extended Data Tables 1, 2.

#### Conservation strategies

We identified global conservation priorities for each tetrapod class using two alternative strategies: strategy 1 prioritized areas containing many threatened species with relatively highly restricted ranges, whereas strategy 2 prioritized areas core to the most range-restricted threatened species globally. We implemented both conservation strategies within the spatial conservation-planning software Zonation^45^ and the R package zonator^46^, using the additive benefit function and the core-area Zonation algorithms for strategies 1 and 2, respectively, at 50-km and 100-km resolutions for threatened reptiles.

The additive benefit function algorithm prioritizes areas by the sum of the proportion of the global range size of all species included in a given grid cell—a quantity similar to weighted species endemism (as defined previously^47^) and endemism richness (as defined^48^). On the basis of this algorithm, cells with many species occurring only in that cell or few other cells receive the highest priority. The core-area Zonation algorithm prioritizes areas by the maximum proportion of the global range size of all species included in a given grid cell: cells including the highest proportions of the ranges of the most range-restricted species are given the highest priority.

Therefore, comparing the two strategies, strategy 1 gives more importance to the number of species within grid cells (that is, more species = a higher summed proportion), potentially at the expense of the single most-range-restricted species globally, which are instead prioritized directly by strategy 2.

Because complementary representation problems such as these spatial prioritizations often have multiple solutions, we ran five iterations of each algorithm used and summarized variation across those.

#### Estimating surrogacy

To assess the degree to which conserving the diversity of threatened species of birds, mammals and amphibians (individually or combined) serves as a surrogate for conserving threatened reptile diversity, we calculated a species accumulation index (SAI) of surrogate effectiveness. The SAI is derived from the comparison of three curves: (1) the ‘optimal curve’ represents the accumulation of the diversity of threatened reptile species when conservation is planned using data for threatened reptiles directly; (2) the ‘surrogacy curve’ represents the accumulation of the diversity of threatened reptile species when conservation is planned using the diversity of threatened species diversity of a different class as a surrogate; and (3) the ‘random curve’ represents the accumulation of the diversity of threatened reptile species when conservation areas are selected at random. We estimated optimal, surrogate and random curves based on each reptile-surrogate combination (birds, mammals and amphibians individually and combined). Using 100 sets of approximately random terrestrial grid-cell sequences allowed us to generate 95% confidence intervals around a median ‘random curve’. In addition, because we ran five iterations of each spatial prioritization algorithm for each tetrapod class, optimal and surrogate curves were also summarized using the median and 95% confidence intervals across the five iterations.

We then derived the quantitative measure of surrogacy as SAI =  (*s* − *r*)/(*o* − *r*), where *s* is the area under the surrogate curve, *r* is the area under the random curve and *o* is the area under the optimal curve. SAI = 1 when the optimal and surrogate curves are the same (perfect surrogacy). It is between 1 and 0 when the surrogate curve lies above the random curve (positive surrogacy), zero when the surrogate and random curves coincide (no surrogacy) and negative when the surrogate curve lies below the random curve (negative surrogacy). We calculated the SAI using R code modified from a previous study^49^. For each reptile–surrogate combination, we report median and 95% confidence intervals across all combinations of optimal, surrogate and random curves (5 target and surrogate curve iterations and 100 random curve iterations).

Although not strictly a measure of surrogacy^25^, we also calculated the spatial congruence (Spearman’s rank correlation, analogous to a previously published approach^9^) of Zonation priorities for each conservation strategy and spatial resolution.

#### Coverage by protected areas

We overlayed protected areas (polygons, categorized as IUCN I–VI from the World Database of Protected Areas^50^) over the ranges of all threatened tetrapods and classified species with ranges completely outside any protected area as unprotected.

#### Phylogenetic diversity

To calculate phylogenetic diversity^15^, we used published time trees of mammals^51^, birds^52^ and amphibians^53^. For reptiles, we combined two time trees: a comprehensive squamate time tree containing 9,755 squamate species, including the species *Sphenodon punctatus*^16^, and a turtle and crocodilian tree containing 384 species^17^. The time trees contain some species lacking genetic data, added by taxonomic interpolation^54^ to maximize taxonomic coverage. In total, we analysed 32,722 tetrapod species including 10,139 reptiles, 5,364 mammals, 9,879 birds and 7,239 amphibians. For squamates, and for turtles and crocodiles, 10,000 fully resolved trees were available. For each group, we randomly sampled 100 trees and combined them to obtain 100 fully resolved reptile time trees, to accommodate for uncertainty. Similarly, we randomly sampled 100 amphibian and 100 mammal time trees over the 10,000 available.

We thoroughly compared the species name mismatches between geographical and phylogenetic data to match synonyms and correct misspelled names. We also imputed species for which the genus (but not the species) was already present in the tree, for example newly described species (262 amphibian, 1,694 bird, 236 mammal and 777 reptile species). Imputed species were randomly attached to a node within the genus subtree. Because polytomies can result in an overestimation of the phylogenetic diversity, we randomly resolved all polytomies using a previously published method^54^ implemented in R code. This procedure was performed 100 times for birds, and one time for each of the 100 amphibian, 100 mammal and 100 reptile time trees. We included 30,778 tetrapod species, each with geographical and phylogenetic data, in the phylogenetic diversity analyses. This total included 6,641 amphibians, 8,758 birds, 5,550 mammals and 9,829 reptiles. For each class, we estimated phylogenetic diversity^14^ for all species and after the removal of threatened species, at 50-km and 100-km resolution. To consider phylogenetic uncertainty (that is, the placement of interpolated species) in phylogenetic diversity calculation for each of the 100 fully resolved trees for each class, we conducted a sensitivity analysis using a previously described method^55^. This method calculates an evolutionary distinctiveness score that (1) increases the total phylogenetic diversity of the clade when including interpolated species and (2) corrects the evolutionary distinctiveness score of species in genera with interpolated species (missing relatives). Following this method, we calculated evolutionary distinctiveness scores^56^ for each cell from the subtree including all species present in the focal cell with the R package caper^57^. For genera with interpolated species, the mean evolutionary distinctiveness score of non-interpolated species was assigned to interpolated species of that genus. For those genera, we computed a second evolutionary distinctiveness score corresponding to the mean evolutionary distinctiveness score of the focal genera (including interpolated species). For species belonging to genera with no interpolated species, the first and second evolutionary distinctiveness scores were identical. Next, we calculated the mean of the two evolutionary distinctiveness scores and reported this value as the evolutionary distinctiveness score of each species. Finally, we computed phylogenetic diversity as the sum of evolutionary distinctiveness scores. Therefore, phylogenetic diversity corresponds to Crozier’s version of phylogenetic diversity^58^, that is, the sum of the branch lengths connecting all members of a species assemblage without the root. Next, we reported median phylogenetic diversity, computed over 100 fully resolved trees for each class. In the figures, cells with fewer than five species were excluded to avoid outliers.

### Reporting summary

Further information on research design is available in the Nature Research Reporting Summary linked to this paper.

## Online content

Any methods, additional references, Nature Research reporting summaries, source data, extended data, supplementary information, acknowledgements, peer review information; details of author contributions and competing interests; and statements of data and code availability are available at 10.1038/s41586-022-04664-7.

### Supplementary information

Supplementary Information This file contains Supplementary Note 1 and Supplementary Tables 1–3.

Reporting Summary

### Source data

Source Data Fig. 1

Source Data Fig. 3

Source Data Fig. 4

## Figures and Tables

**Extended Data Table 1:**
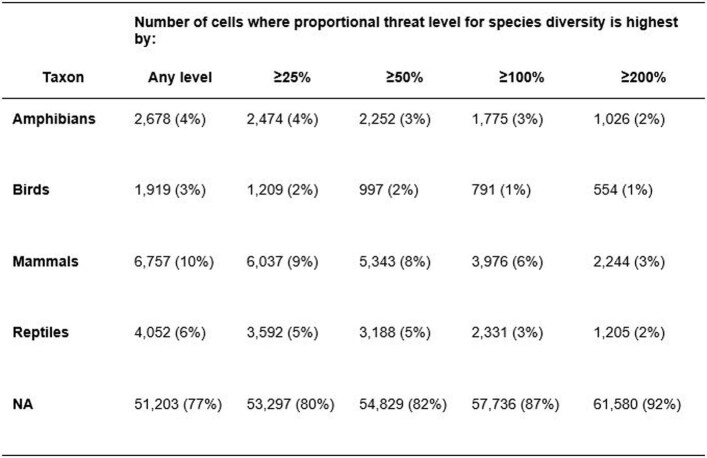
Sensitivity analysis for proportional threat level differences

Influence of choice of threshold for proportional threat level difference between the most and second most proportionately threatened class on the absolute and relative number of grid cells identified as disproportionately threatened for species diversity across the four tetrapod classes. Provided in brackets are the percentage of all global terrestrial cells (71,605 in total) that are either attributed to the given class or assigned NA if no class is identified as disproportionately threatened using the corresponding threat level. Note that in the main text, we report results for the 100% or more threat level.

**Extended Data Table 2:**
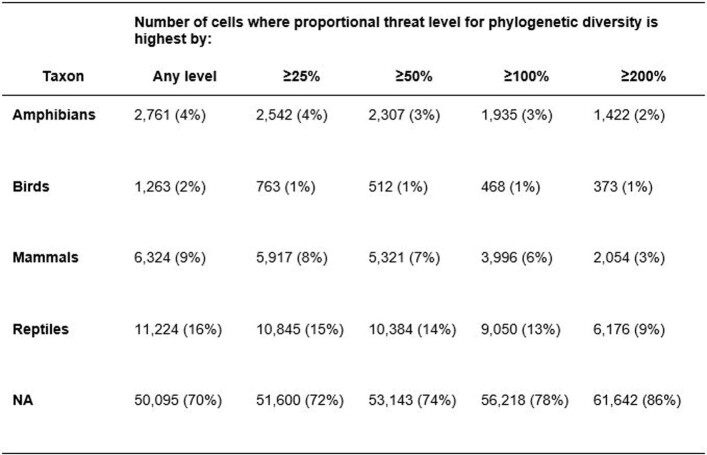
Sensitivity analysis for proportional threat of phylogenetic diversity loss differences

Influence of choice of threshold for proportional threat level difference between the most and second most proportionately threatened class on the absolute and relative number of grid cells identified as disproportionately threatened for phylogenetic diversity across the four tetrapod classes. Provided in brackets are the percentage of all global cells (71,605 in total) that are either attributed to the given class or assigned NA if no class is identified as disproportionately threatened using the corresponding threat level. Note that in the main text, we report results for the 100% or more threat level.

**Extended Data Table 3:**
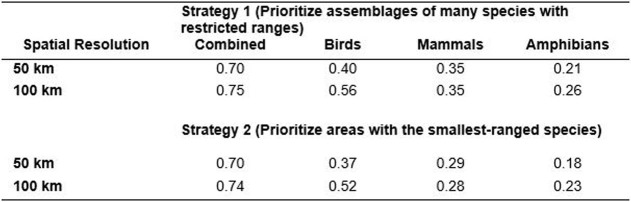
Spatial congruence of Zonation priorities between reptiles and birds, mammals, and amphibians

Shown are Spearman’s rank correlation coefficients among priority ranks for each conservation strategy and spatial resolution examined. Note that spatial congruence is not a strict metric of surrogacy because it does not address complementarity among areas^24^. “Combined” refers to birds, mammals, and amphibians combined.

**Extended Data Table 4:**
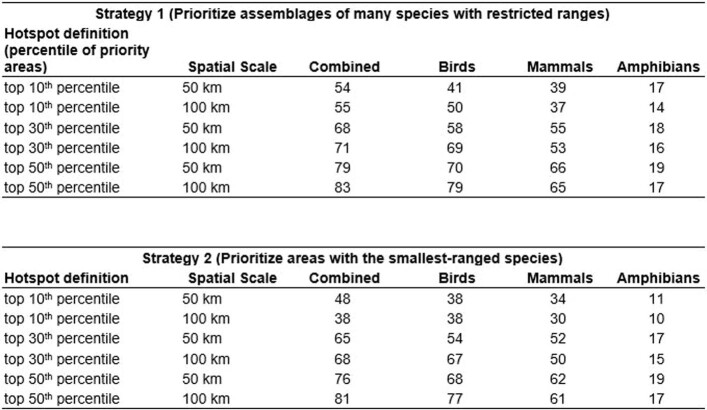
Percentage overlap in highest conservation priority areas between threatened reptiles and threatened birds, mammals, and amphibians, both individually and combined

Highest conservation priority areas were defined as the top 10th, 30th or 50th percentile areas ranked in our systematic spatial conservation planning analyses for the respective taxon. Overlap was calculated as the number of highest priority cells shared between taxa divided by the total number of terrestrial cells with threatened reptiles, expressed as a percentage. Percentage overlap is shown at 50 km and 100 km grid cell resolutions.

## Data Availability

Taxonomic data for reptiles were from the Reptile Database (http://www.reptile-database.org/index.html). All spatial and tabular data for the tetrapod analyses are permanently available at https://transfer.natureserve.org/download/Publications/Global_Reptiles/. Trees used for the phylogenetic diversity analyses are available at Zenodo (https://zenodo.org/record/5974891). In addition, assessment data, including range maps, for all tetrapods are publicly available on the IUCN Red List of Threatened Species website (https://iucnredlist.org). Occasionally, where a species may be threatened because of over-collection, sensitive distribution information is not publicly available. Protected areas boundaries were from the World Database of Protected Areas (https://www.protectedplanet.net). Source data are provided with this paper.

## Code availability

Python scripts used for the spatial analyses are permanently available at https://transfer.natureserve.org/download/Publications/Global_Reptiles/. No code was used for the Fisher’s exact tests, which were performed in Excel and available with the tabular data at https://transfer.natureserve.org/download/Publications/Global_Reptiles/. Code used for the phylogenetic diversity, areas of disproportionate threat and surrogacy analyses are available at Zenodo (https://zenodo.org/record/5974891).
