# Supplementary material for: A global reptile assessment highlights shared conservation needs of tetrapods
Source: Nature. 2022 Apr 27;605(7909):285–90. doi: 10.1038/s41586-022-04664-7 (PMC9095493; doi:10.1038/s41586-022-04664-7)
Supplement: Supplementary file 1 — This file contains Supplementary Note 1 and Supplementary Tables 1–3. [file 41586_2022_4664_MOESM1_ESM.pdf]

nature portfolio

https://doi.org/10.1038/s41586-022-04664-7

Supplementary information

A global reptile assessment highlights shared conservation needs of tetrapods

In the format provided by the authors and unedited

Global Reptile Assessment Shows Commonality of Tetrapod Conservation Needs

By Cox, Young, et al.

SUPPLEMENTARY INFORMATION

Supplementary Note 1

List of Assessors

The following people contributed to the assessment of at least one reptile species used in this study. Assessors that are also authors either provided substantial unpublished datasets, hosted assessment workshops and contributed to numerous assessments, and/or served as a reptile Red List Authority.

S. Abdala, C. Abrahão, M. Acevedo, N. Achyuthan, V. Acosta Chaves, K. Adolphs, R. Aengals, L. Afuang, I. Agarwal, A. Agasyan, A. Aguayo, R. Aguayo, C. Aguilar, N. Rakotondrazafy, R. Ajtic, G. Akani, F. Akarsu, A. Al Johany, M. Al Jumaily, K. Al Rasbi, G. Alexander, A. Allison, P. Almeida-Santos, A. Almendáriz, J. Alonso, A. Aloufi, Y. Amapou, V. Amaral de Menezes, Y. Amepou, A. Amey, Z. Amr, N. Ananjeva, C. Anderson, S. Anderson, C. Andrén, F. Andreone, A. Andriamazava, R. Andriantsimanarilafy, N. Angeli, M. Anjeriniaina, R. Antelo, R. Antolin, S. Anuar, J. Aparicio, D. Ardon, A. Argôlo, D. Ariano-Sánchez, E. Arida, A. Ariefiandy, J. Arredondo, A. Arteaga-Navarro, V. Arzamendia, M. As-singkily, S. Astrongatt, S. Augros, M. Auliya, C. Austin, A. Avci, L. Avila, R. Ávila, T. Avila-Pires, R. Avilés, F. Ayala, M. Azmi, E. Baard, V. Bachraz, S. S. Bafti, S. Baha El Din, R. Bain, P. Baker, S. Balaguera-Reina, E. Ballesteros, K. Balta Abadie, M. J. Bamford, G. Baorong, N. Baptista, R. Baptista de Oliveira, F. Barbo, C. Barrio-Amorós, M. Barros, L. Bassetti, Y. Bataus, M. Bates, A. Batista, J. Bauder, A. Bauer, J. Bayliss, F. Becker, M. Behangana, S. Behbehani, N. Behler, A. Belouskova, D.

Bennett, J. Beraduccii, R. Bérnils, E. Bevan, M. Bezuijen, S. Bhattarai, J. Bi, T. Blanck, C. Block, B. Bock, M. Böhm, W. Böhme, W. Bolívar, F. Bonilla, P. Bora, B. Borczyk, M. Borges-Martins, D. M. Borges-Nojosa, L. Borkin, A. Borzee, M. Botejue, R. Bour, E. Bourguet, A. Bouteiller, P. Bowles, R. Boycott, K. Bradley, L. Brady, W. Branch, R. Bressan, M. Breuil, J. Brito, D. Broadley, R. Brown, M. Bruton, S. Buckland, S. Buckner, E. Buongermini, M. Burger, R. L. Burke, F. Burton, S. Busais, J. Buskirk, L. Bustamante, M. Cabrera, P. Cacciali, A. Caccone, B. Cai, J. R. Caicedo, M. Calderón, B. Camera, J. Campbell, Z. Campos, C. Canales Ramirez, L. Canseco-Márquez, A. Captain, R. Cardoso Vieira, A. Carpenter, S. Carranza, S. Carreira, R. Carreras De León, P. Casale, F. Castro, A. Catenazzi, G. Catt, L. Cayot, S. Cechin, L. Ceríaco, E. H. Chan, T. Chan-Ard, Y. Chapeta, D. Chapple, G. Chaves, J. C. Chavez, G. Chavez, J. Chenga, M. Cheylan, Z. Chifundera, J. Chippaux, M. Chirikova, L. Chirio, B. Choudhury, S. Chowfin, C. Ciofi, D. F. Cisneros-Heredia, S. Clayson, N. Clemann, D. Cogalniceanu, H. Cogger, F. Colacicco, N. Cole, G. Colli, G. Colosimo, T. Colston, X. Combrink, W. Conradie, C. Cortez, C. Corti, G. Costa, H. Costa, M. Cota, P. Couper, T. Courtney, M. Coutinho, M. Cowan, N. Cox, M. Craig, J. Crnobrnja-Isailovic, P. Crochet, A. Crottini, F. Cruz, M. Cunningham, T. da Costa, O. da Cunha, C. F. da Rocha, J. Daltry, P. Danaisawat, A. Das, I. Das, J. Dawson, J. Daza, N. D'Cruze, L. de Alencar, S. de Alwis Goonatilake, S. de Barcelos Ribeiro, M. A. de Freitas, C. De Haan, J. de Leon, M. de Lourdes Vázquez-Cruz, G. de Moura, K. de Queiroz, A. de Silva, R. De Silva, A. de Villiers, V. Deepak, M. Dehling, M. del Carmen Blázquez, M. del Rosario Castañeda, E. del Valle, E. Delima, A. Demegillo, M. Deuss, K. Deuti, N. Dewhurst, M. Dewynter, D. Di Pietro, T. Diagne, S. Díaz, C. Dickman, A. Diesmos, M. Diesmos, M. Dimaki, J. Dimalibot, L. Dirksen, T. Doan, M. Dorcas, I. Doronin, P. Doughty, J. Draque, G. Drummond, P. Duengkae, T. Dujsebayeva, T. Duval, Q. Dwyer, A. Echternacht, T. Edwards, D. Egan, E. Eid, C. Eisemberg, E. H. El Mouden, R. Ellis, J.

Els, R. Elsey, D. Embert, J. Emery, E. Eniang, P. Espejo, A. Espinosa-Blanco, R. Espinoza, A. Estrades, E. Etchepare, H. Farooq, A. Feldman, J. Fenker, A. Fenner, D. Ferguson, V. L. Ferreira, C. Figuerola, M. Firoz Ahmed, R. Fisher, L. Fitzgerald, M. Flecks, O. Flores-Villela, J. Fong, A. Fong, S. Ford, F. França, F. Franco, S. French, U. Fritz, D. Frost, D. Gabadage, H. Gadsden, G. Gagliardi, M. Gaiarsa, G. Gaikhorst, N. Gallego-Garcia, S. Ganesan, S. R. Ganesh, M. Garcia, A. García Rodríguez, J. García-Pérez, A. Gardner, C. Garin, J. Gatus, M. Gaulke, P. Geniez, G. Gentile, A. Georges, G. Gerber, J. Gerlach, H. Ghaffari, A. Ghosh, J. P. Gibbs, R. Gibson, J. Gil-Escobedo, G. Gillespie, A. Giraudo, V. Giri, M. Girondot, F. Glaw, M. Goder, N. Goicoechea Rodriguez, S. Goldberg, E. Golynsky, R. Gomès, R. Gomes Faria, L. Gonzales, J. C. Gonzalez, E. Goode, A. B. C. Goode, D. Gower, T. Grant, K. Gray, E. Greenbaum, M. Greenlees, C. Grieco, R. Griffiths, L. Grismer, N. Guillerault, M. Guinea, S. Gunawardene, J. Guntoro, X. Guo, P. Guo, P. Gutierrez, G. L. Gutiérrez de la Cruz, P. Gutiérrez-Cárdenas, I. J. Hagen, J. Hallermann, A. Hamidy, A. Hamilton, P. Hamilton, G. Hammerson, k. Hare, P. Harlow, M. Harvey, C. Hasbún, S. Hathaway, S. Hauser, O. Hawlitschek, I. Haxhiu, A. Hector, B. Hedges, E. Hema, R. Henderson, E. Henningheim, R. Hitchmough, A. I. Hladki, H. Hoang, R. Hobson, M. Hofmeyr, B. Hollingsworth, M. Hoogmoed, B. Horne, P. Horner, J. Horrocks, C. Hoskin, R. How, K. Howell, S. Hraoui-Bloquet, S. Huang, M. Hutchinson, M. Ibáñez, R. Ibáñez, J. Imansyah, S. Inchaustegui, I. Ineich, R. Inger, Y. Ioannidis, S. Isberg, D. Iskandar, N. Jacobsen, M. Jallow, C. Jaramillo, D. Jayasekara, D. Jelic, H. Jenkins, R. Jenkins, A. Jerez, T. Jessop, X. Ji, H. Jiang, J. Jiang, O. Jiménez, U. Joger, R. Joglar, J. Johnny, J. Johnson, C. Jones, J. Jose, R. Jose, H. Jourdan, J. O. Juvik, J. Kabigumila, F. Kacoliris, M. Kamrul Hasan, M. Kamsi, D. Kandambi, S. Kannishka, A. Karl, B. Karlsdottir, M. Kartje, S. Karunarathna, Y. Kasaka, Y. Kaska, R. Kawashita-Ribeiro, U. Kaya, J. Kelly, S. Keogh, T. Keswick, M. M. Khan, M. Khan, M. Khan, N. Kidera, M. Kiefer, R. Kiester, C. Knapp, C. Koch, A. Koch, S. Koenig,

G. Köhler, P. Kok, P. Kornacker, L. Krecsák, G. Kuchling, N. U. Kulkarni, K. Kumar, P. Kumari de Silva, Y. Kumlutas, C. Kusamba, M. Kusrini, M. Kyek, E. La Marca, R. Labanowski, A. Lagrange, W. Lamar, M. Landestoy, J. Lang, R. Langstroth, A. Larriera, M. Lau, P. Lavin, M. LeBreton, M. Ledesma, J. Lee, N. LeGrand Gonwouo, E. Lehr, D. Leite, M. Leite Oliveira, M. Lettink, T. Leuteritz, S. Lewis, P. Li, R. Lilley, A. Lima, S. Lin, C. Linkem, P. Lintott, C. Lippai, S. Litvinchuk, A. Llobet, R. Lloyd, S. Loader, A. Lobo, G. Lobos, D. Loebmann, V. Loehr, M. Logan, M. López-Luna, S. Lötters, L. Luiselli, L. Luiz, V. Lukoschek, M. Lundberg, I. Luque, V. Luu, P. Lymberakis, S. Macdonald, R. Macey, A. MacLeod, M. Madala, M. Madawala, W. Magnusson, D. L. Mahler, D. Mallon, C. Malone, P. Malonza, R. Mandujano, R. Maneyro, C. Manolis, U. Manthey, J. Marais, Y. Marambio, B. Maritz, O. Marques, C. Márquez, L. Marquez, R. Márquez, A. Martinez, I. Martinez Solano, F. Martínez-Freiría, M. Martins, B. Maryan, D. F. Maryon, G. Maschio, G. Masterson, J. Mateo, L. Mavoungou, G. Mayer, T. Mazuch, T. McCormack, M. McCoy, S. McCoy, J. McCranie, P. McDonald, J. McGuire, J. Measey, K. Mebert, S. Meiri, C. Melendez Torres, J. Mella, D. Melnikov, J. Melville, F. Mendoza-Quijano, M. Menegon, V. P. Mercado, S. Métrailler, A. Meyer, D. Michael, P. Micucci, A. H. Miller, H. Milligan, K. Milto, E. Miranda, N. Mitchell, R. A. Mittermeier, E. Mizsei, A. Mobaraki, S. Mohammed, P. Mohapatra, D. Moll, E. Moll, P. Molur, S. Molur, O. Monson, R. Montero, C. Montgomery, O. Monzón, R. Mootoocurpen, M. Morales-Betancourt, J. Morales-Mávil, W. Moral-Uribina, J. Moravec, R. Moreno, C. Moritz, T. Mott, A. M. Disi, L. F. Mouton, C. Msuya, D. Mulcahy, F. Muniz, M. Munkhbaatar, K. Munkhbayar, G. Munoz, A. Muñoz, A. Muñoz-Alonso, J. Murphy, C. Murthy, R. Mwaya, S. Narayanan, R. Nazarov, K. Nelson, H. Nettmann, W. Ngalason, H. N. Ngo, N. S. Nguyen, T. Nguyen, J. M. Ngwava, B. Niagate, K. Nicholson, G. Nilson, C. Nogueira, S. Nouira, H. Nunez, J. Nunez, D. Nuridjanov, M. Oliveira, R. Oliveira, P. Oliver, K.O. Chan, N. Orlov, V. Orlova, A. Ortega, J. C. Ortiz, M. O'Shea, H. Ota, P. Oubotar,

L. Outlaw, N. Pacheco-Hoyos, V. Páez, L. M. Paguntalan, S. Pal, R. Palomo-Ramos, R. Paltridge, N. Panitvong, T. Papenfuss, F. Parker, S. Pasachnik, P. Passos, G. Patterson, O. Pauwels, P. Pawangkanan, C. Payne, N. Pelegrin, J. Penner, D. Pereira, N. Perera, J. Perez, P. Perez, R. Perez Maciel, V. Pérez Mellado, F. Petrozzi, S. Phimmachak, D. Pietersen, C. Piña, R. Platenberg, K. Platt, S. Platt, J. Pleguezuelos, A. Plumptre, R. Podloucky, S. Poe, C. C. Pomaro, P. Ponce-Campos, G. Pontes, L. Porras, R. Powell, G. Powney, M. Prabhu, K. Prasad Limbu, P. Praschag, D. Proetzel, A. Prudente, A. Puente-Rolón, D. Purwandana, V. Pushpamal, E. Quah, G. Quintero Díaz, A. Quiroz Rodriguez, N. Rabibisoa, R. Radder, J. Rafanomezantsoa, S. C. Rahman, A. Raimundo, D. Rakotomalala, H. Rakotondravony, J. Ramanamanjato, M. Ramesh, M. P. Ramirez, C. Ramirez Peralta, H. Randriamahazo, C. Randrianantoandro, H. Randrianasolo, J. Randrianiriana, H. Randrianizahana, D. Rao, A. Raselimanana, A. Rasmussen, F. Ratsoavina, C. Raxworthy, M. Read, R. Recoder, S. Reilly, J. Ren, J. M. Renjifo, A. Restrepo, C. Reyes-Puig, G. Reynolds, R. G. Reynolds, V. Reynoso, A. Rhodin, L. Ribeiro, M. A. Ribeiro Júnior, S. Richards, N. Richman, E. Rico, J. Ríos-Arana, E. Riservato, G. Rivas, R. C. Rivera-Arroyo, A. Riyanto, S. Roberton, P. Robertson, E. Robsomanitrandrasana, G. Rodda, D. Rödder, M. Rödel, J. Rodrigues dos Santos Silva, C. Rodriguez, L. Rodriguez, J. Rodríguez, A. Romano, P. Roopa, W. Roosenburg, R. G. Rosell-Ambal, P. Rosen, P. Ross, S. Roussos, A. Roy, K. Ruhomaun, M. Ruiz de Gamboa, N. Rusli, E. Rustamov, G. Saborío, R. Sadek, R. Sadlier, I. Safari, H. Salinas Matus, N. Sallabery, D. Samarasinghe, P. Samarawickrama, M. Sanchez, J. C. Sánchez, K. Sanders, C. Sanderson, G. Santos-Barrera, C. Sardinha, M. Sasa, P. Sá-Sousa, T. Sattorov, N. Sawant, R. Sawaya, D. Scantlebury, W. Schargel, U. Schepp, F. Schmidt, B. Schmidt, A. Schmitz, H. Schneider, S. Schoppe, N. Scott, G. Scrocchi, J. Sears, G. Segniagbeto, J. Segovia, A. Seijas, M. Sevinç, G. Shankar, M. Sharifi, G. Shea, C. Shepherd, A. Shestopal, H. Shi, L. Shi, Y. Shin, R. Shine, M. Shirley, M. Shobrak, B. Shwedick, A. Silveira, B. Simpson, R. Sindaco, S.

Singh, P. Siroski, A. Sivaruban, T. Slimani, N. Smaniotto, K. Soe Lwin, G. Solmu, A. Solórzano, R. Somaweera, S. Sondhi, P. Soorae, S. Spawls, R. Sreekar, B. Srinivasulu, C. Srinivasulu, P. Stafford, C. Stanford, A. Staniewicz, B. Sterijovski, J. Stipala, C. Strüssmann, B. Stuart, A. Stubbs, R. Stuebing, J. Suárez, M. Sumontha, J. Sunyer, M. Suraj, S. Sweet, E. Sy, M. Szabolcs, C. Tala, O. Tallowin, G. Tampos, W. Tapia, E. Taskavak, V. Tatayah, R. Teale, L. Tedeschi, Y. Tempsiripong, K. Terbish, W. Thaksintham, S. Thakur, A. A.T. Amarasinghe, R. Thomas, S. Thomson, N. Thy, C. Tilbury, R. Timmins, M. Tinôco, M. Tiwari, B. Tjaturadi, M. Tognelli, V. Tok, K. Tolley, P. Tolson, L. Tomovic, S. Torquato da Silva, C. Torres, C. Toudonou, J. Townsend, J. Trape, P. Trivalarait, S.-Y. Tsai, S. Tshewang, A. Tucker, B. Tuniyev, S. Tuniyev, O. Turkozan, A. Turner, M. Ubeda, I. Ugurtas, V. Uhlig, K. Ukuwela, N. Urbina, N. Üzüm, R. Valadão, J. Valencia, L. Valentine, P. Valladares, S. van Barneveld, G. van Buurt, M. P. van den Burg, P. P. van Dijk, M. van Schingen, M. van Weerd, D. van Winkel, E. Vanderduys, J. Vargas Álvarez, R. Vasconcelos, M. Vaughn, W. Vaz, P. Vaz Pinto, J. Vazquez Díaz, L. Vega, J. Velasco, M. Velasco, A. Velasco, D. Vencatasamy, M. Vences, P. Venegas, M. Venz, L. Verburgt, L. Verrastro Viñas, M. Veselý, P. Victoriano, M. Vidal, D. Vidanapathirana, S. Vijayakumar, S. Vinke, T. Vinke, G. Vogel, M. Vogrin, R. Vogt, R. Vyas, P. Wagner, P. Walker, R. Walker, B. Wallace, V. Wallach, T. Waller, J. Wang, L. Wang, Y. Wang, Y. Wang, J. T. Wangyal, E. Wapstra, O. Wearn, J. Webb, J. Weeber, F. Weinsheimer, F. Werneck, Y. Werner, A. Westerström, A. Whitaker, M. Whiting, T. Wibbels, M. Wickramasinghe, N. Wickramasinghe, N. Wijethilaka, S. Wikramanyake, J. Wilkinson, J. Williams, T. Wilms, S. Wilson, B. Wilson, L. Wilson, P. Wilson, G. Winck, D. Wingate, G. Wogan, J. Woinarski, R. C. Wood, A. Woodward, E. Wostl, S. Wren, X. Wu, M. Wynn, G. Xiang, Q. Xiang, F. Xie, Y. Xie, M. Yánez-Muñoz, J. Yang, C. Yeniyurt, H. Zaher, T. Zamin, G. Zamora, E. Zarza-Franco, A. Zassi-Boulou, W. Zhao, W. Zhou, Z. Zhou, J. Zichy-Woinarski, T. Ziegler, G. Zug.

Supplementary Table 1.

In-person and remote workshops undertaken as part of the Global Reptile Assessment.

Year	Region	Assessment Type
2004	Mediterranean	Workshop
2005	Mexico	Workshop
2006	Northern and Central Europe	Workshop
2006	Seychelles	Remote assessment
2006	North America	Remote assessment
2008	Caucasus, Iran, and Iraq	Workshop
2008	Philippines	Workshop
2009	Caribbean (iguanas)	Workshop
2011	Madagascar	Workshop
2011	Comoros	Remote assessment
2011	China and Southeast Asia (snakes)	Workshop
2011	Oceania	Workshop
2012	Arabian Peninsula	Workshop
2012	Socotra	Remote assessment
2012	Central America	Workshop
2012	West Africa	Workshop
2012	Cabo Verde	Remote assessment
2012	Brazil (snakes)	ICMBio workshops (two held in 2012)
2013	Northwestern Andes	Workshop
2013	Western Ghats	Workshop
2014	New Guinea	Workshop
2014	East Africa	Workshop
2014	Northern Andes	Workshop
2014	Southeastern South America	Workshop (excluded Brazil)
2014	Widespread species of the Amazon Basin, Guiana Shield, and Venezuela	Workshop
2015	Chile	Workshop
2015	Caribbean	Workshop
2015	Cuba	Remote assessment
2015	Venezuela	Workshop
2016	Aruba, Bonaire and Curaçao	Remote assessment
2016	Central Asia	Workshop
2016	Japan	Remote assessment
2017	Western Australia	Workshop
2017	Eastern Australia	Workshop
2018	Southeast Asia (lizards)	Workshop
2018	China (lizards)	Workshop
2019	Korean Peninsula	Remote assessment
2019	Central and Southern Africa	Workshop
2019	Wallacea	Workshop

Year	Region	Assessment Type
2019	Mainland South Asia	Workshop
2019	Pakistan	Remote assessment
2019	Sri Lanka	Workshop
2010/2017	New Caledonia	IUCN workshop/national assessment workshop
2013/2014	Brazil (lizards)	ICMBio workshops
2013/2018	South Africa	SANBI workshops
2016-2019	Mascarenes	Remote assessment
2018-2019	New Zealand	Hybrid workshop/remote assessment
2017-2019	Horn of Africa	Hybrid remote assessment/meeting

Supplementary Table 2.

Summary of Red List Categories for all families of reptiles. See Methods for calculation of best estimate of percent threatened, which does not include EX or EW species.

Order/Suborder/Family	LC	NT	DD	VU	EN	CR	EW	EX	Total	% DD	% Threatened
Crocodylia	12			5		7			24	0.0%	50.0%
Crocodylia	12			5		7			24	0.0%	50.0%
Alligatoridae	7					1			8	0.0%	12.5%
Crocodylidae	5			5		5			15	0.0%	66.7%
Gavialidae						1			1	0.0%	100.0%
Rhynchocephalia	1								1	0.0%	0.0%
Rhynocephalia	1								1	0.0%	0.0%
Sphenodontidae	1								1	0.0%	0.0%
Squamata	6146	532	1485	557	724	350	2	24	9820	15.1%	19.6%
"Lizards"	3905	386	800	392	545	266	2	20	6316	12.7%	21.9%
Anguimorpha	103	6	28	11	42	8			198	14.1%	35.9%
Anguidae	55	3	17	8	28	7			118	14.4%	42.6%
Anniellidae	1				1				2	0.0%	50.0%
Helodermatidae	1	1							2	0.0%	0.0%
Lanthanotidae					1				1	0.0%	100.0%
Shinisauridae					1				1	0.0%	100.0%
Varanidae	44	2	10	2	9	1			68	14.7%	20.7%
Xenosauridae	2		1	1	2				6	16.7%	60.0%
Dibamia	7	2	10	1	3	1			24	41.7%	35.7%
Dibamidae	7	2	10	1	3	1			24	41.7%	35.7%
Gekkota	994	109	240	117	149	91	1	4	1705	14.1%	24.4%
Carphodactylidae	22	3		2	2	2			31	0.0%	19.4%
Diplodactylidae	89	14	6	13	17	9		1	149	4.0%	27.5%
Eublepharidae	17	1	3	3	9	4			37	8.1%	47.1%
Gekkonidae	635	64	201	84	73	57	1	3	1118	18.0%	23.4%
Phyllodactylidae	81	9	9	6	7	5			117	7.7%	16.7%
Pygopodidae	37	1	3	1	3				45	6.7%	9.5%
Sphaerodactylidae	113	17	18	8	38	14			208	8.7%	31.6%
Iguania	1121	126	162	129	168	68		4	1778	9.1%	22.6%
Agamidae	353	20	61	28	25	10			497	12.3%	14.4%
Chamaeleonidae	79	39	10	22	42	10			202	5.0%	38.5%
Corytophanidae	9								9	0.0%	0.0%
Crotaphytidae	9			1	2				12	0.0%	25.0%
Dactyloidae	215	36	39	20	48	22			380	10.3%	26.4%
Hoplocercidae	11			4	2				17	0.0%	35.3%
Iguanidae	8	3	2	11	12	8		1	45	4.4%	73.8%
Leiosauridae	20	1	4	2	3	2			32	12.5%	25.0%
Liolaemidae	207	12	22	19	21	8			289	7.6%	18.0%
Opluridae	7								7	0.0%	0.0%
Phrynosomatidae	102	7	7	9	6	1			132	5.3%	12.8%
Polychrotidae	5		1	1					7	14.3%	16.7%
Tropiduridae	96	8	16	12	7	7		3	149	10.7%	20.0%

Order/Suborder/Family	LC	NT	DD	VU	EN	CR	EW	EX	Total	% DD	% Threatened
Lacertoidea	574	56	125	33	76	27		2	893	14.0%	17.8%
Amphisbaenidae	98	7	39	6	14	6			170	22.9%	19.8%
Bipedidae	3								3	0.0%	0.0%
Cadeidae	2								2	0.0%	0.0%
Gymnophthalmidae	141	14	47	13	30	5			250	18.8%	23.6%
Lacertidae	220	23	26	8	25	12			314	8.3%	15.6%
Rhineuridae	1								1	0.0%	0.0%
Teiidae	106	12	10	6	7	4		2	147	6.8%	12.6%
Trogonophidae	3		3						6	50.0%	0.0%
Scinciformata	1106	87	235	101	107	71	1	10	1718	13.7%	18.9%
Cordylidae	58	3	4	2	1				68	5.9%	4.7%
Gerrhosauridae	26	3	1	6	2			1	39	2.6%	21.6%
Scincidae	1009	79	222	90	103	71	1	9	1584	14.0%	19.5%
Xantusiidae	13	2	8	3	1				27	29.6%	21.1%
Serpentes (“Snakes”)	2241	146	685	165	179	84		4	3504	19.5%	15.2%
Acrochordidae	3								3	0.0%	0.0%
Aniliidae	1								1	0.0%	0.0%
Anomalepididae	7		11						18	61.1%	0.0%
Anomochilidae	1		2						3	66.7%	0.0%
Atractaspididae	63	1	7						71	9.9%	0.0%
Boidae	41	8	2	3	5	1			60	3.3%	15.5%
Bolyeridae				1				1	2	0.0%	100.0%
Calamariidae	41		37	4	4	4			90	41.1%	22.6%
Colubridae	540	24	114	25	23	10			736	15.5%	9.3%
Cylindrophiidae	2	1	7	1		1			12	58.3%	40.0%
Dipsadidae	452	32	154	33	41	24		2	738	20.9%	16.8%
Elapidae	268	15	51	16	9	2			361	14.1%	8.7%
Gerrhopilidae	3		12		1	1			17	70.6%	40.0%
Grayiidae	4								4	0.0%	0.0%
Homalopsidae	20	1	23	2	2				48	47.9%	16.0%
Lamprophiidae	69		9		1				79	11.4%	1.4%
Leptotyphlopidae	72	2	35	3	2	4			118	29.7%	10.8%
Loxocemidae	1								1	0.0%	0.0%
Natricidae	148	11	42	11	12	4			228	18.4%	14.5%
Pareidae	14	1	4						19	21.1%	0.0%
Prosymnidae	14				1	1			16	0.0%	12.5%
Psammophiidae	47	1		1					49	0.0%	2.0%
Pseudoxenodontidae	8		4						12	33.3%	0.0%
Pseudoxyrhophiidae	52	12	7	11	8	2			92	7.6%	24.7%
Pythonidae	24	4	6	6	1				41	14.6%	20.0%
Tropidophiidae	10	4	2	6	9	6			37	5.4%	60.0%
Typhlopidae	120	6	105	7	16	7		1	262	40.1%	19.2%
Uropeltidae	12	2	20	4	12	6			56	35.7%	61.1%
Viperidae	193	20	27	30	32	10			312	8.7%	25.3%
Xenodermidae	9	1	2	1		1			14	14.3%	16.7%
Xenopeltidae	2								2	0.0%	0.0%
Xenophiidae			2						2	100.0%	NA
Testudines	97	39	22	61	56	69		7	351	6.3%	57.8%
Testudines	97	39	22	61	56	69		7	351	6.3%	57.8%
Carettochelyidae					1				1	0.0%	100.0%
Chelidae	27	9	8	4	4	5			57	14.0%	26.5%

Order/Suborder/Family	LC	NT	DD	VU	EN	CR	EW	EX	Total	% DD	% Threatened
Cheloniidae			1	2	1	2			6	16.7%	100.0%
Chelydridae	1	1		3					5	0.0%	60.0%
Dermatemydidae						1			1	0.0%	100.0%
Dermochelyidae				1					1	0.0%	100.0%
Emydidae	16	9	5	15	9	1			55	9.1%	50.0%
Geoemydidae	10	9	2	4	20	26			71	2.8%	72.5%
Kinosternidae	19	6		2		2			29	0.0%	13.8%
Pelomedusidae	13	1	4		1				19	21.1%	6.7%
Platysternidae						1			1	0.0%	100.0%
Podocnemididae				4	1	3			8	0.0%	100.0%
Testudinidae	6	4		17	12	19		7	65	0.0%	82.8%
Trionychidae	5		2	9	7	9			32	6.3%	83.3%
**Total**	**6256**	**571**	**1507**	**623**	**780**	**426**	**2**	**31**	**10,196**	**14.8%**	**21.1%**

Supplementary Table 3.

Threatened (Critically Endangered, Endangered, and Vulnerable) tetrapods with ranges that do not overlap with the ranges of any other threatened tetrapod. Analysis performed using range maps developed as part of the extinction risk assessment process.

Birds

Acrocephalus aequinoctialis	*Edolisoma nesiotis*	*Oreomystis bairdi*
Acrocephalus caffer	*Elaenia ridleyana*	*Palmeria dolei*
Acrocephalus familiaris	*Erythropitta splendida*	*Paroreomyza montana*
Acrocephalus longirostris	*Eunymphicus uvaeensis*	*Passer hemileucus*
Acrocephalus rehsei	*Fringilla polatzeki*	*Petroica dannefaerdi*
Acrocephalus rimitarae	*Gallinula comeri*	*Petroica traversi*
Acrocephalus taiti	*Geospiza acutirostris*	*Pinaroloxias inornata*
Acrocephalus vaughani	*Geospiza pauper*	*Pomarea iphis*
Aerodramus sawtelli	*Geospiza propinqua*	*Pomarea mendozae*
Alopecoenas erythropterus	*Geospiza septentrionalis*	*Pomarea nigra*
Alopecoenas rubescens	*Hemignathus wilsoni*	*Pomarea whitneyi*
Anas aucklandica	*Hemiphaga chathamensis*	*Pseudonestor xanthophrys*
Aphrastura masafucrae	*Junco insularis*	*Ptilinopus chalcurus*
Atlantisia rogersi	*Lewinia muelleri*	*Ptilinopus chrysogaster*
Branta sandvicensis	*Loxioides bailleui*	*Ptilinopus huttoni*
Charadrius sanctaehelenae	*Loxops caeruleirostris*	*Ptilinopus insularis*
Chasiempis ibidis	*Loxops coccineus*	*Rowettia goughensis*
Chasiempis sandwichensis	*Magumma parva*	*Sephanoides fernandensis*
Chasiempis sclateri	*Manucerthia mana*	*Sittiparus owstoni*
Chlorodrepanis flava	*Mayrornis schistaceus*	*Symposiachrus ateralbus*
Chlorodrepanis stejnegeri	*Mimus trifasciatus*	*Telespiza cantans*
Coccyzus ferrugineus	*Myadestes obscurus*	*Todiramphus gambieri*
Coenocorypha pusilla	*Myadestes palmeri*	*Todiramphus godeffroyi*
Cyanoramphus forbesi	*Myiagra cervinicolor*	*Todiramphus ruficollaris*
Cyanoramphus unicolor	*Nesospiza acunhae*	*Vini peruviana*
Drepanis coccinea	*Nesospiza questi*	*Vini stepheni*
Ducula aurorae	*Nesospiza wilkinsi*	*Vini ultramarina*
Ducula galeata	*Nesotriccus ridgwayi*	*Zapornia atra*

Mammals

Echymipera davidi	*Peromyscus dickeyi*	*Pteropus rodricensis*
Lepus insularis	*Peromyscus pseudocrinitus*	*Rattus nikenii*
Mogera uchidai	*Peromyscus sejugis*	*Sorex pribilofensis*
Peromyscus caniceps	*Phalanger alexandrae*	

Reptiles

Alsophis antiguae	*Flexiseps valhallae*	*Oligosoma fallai*
Anolis nelsoni	*Gongylomorphus bojerii*	*Phymaturus mallimaccii*
Anolis villai	*Lepidodactylus tepukapili*	*Plestiodon longirostris*

Aristelliger nelsoni
Aspidoscelis martyris
Bavayia exsuccida
Carinascincus palfreymani
Correlophus belepensis
Cryptoblepharus caudatus
Ctenosaura nolascensis
Cubophis brooksi
Liolaemus famatinae
Liolaemus morenoi
Lygodactylus insularis
Mabuya cochonae
Macrovipera schweizeri
Nactus coindemirensis
Nactus serpensinsula
Podarcis gaigeae
Podarcis levendis
Podarcis milensis
Uta encantadae
Uta lowei
Uta palmeri
Uta tumidarostra

Amphibians

Atelognathus patagonicus
Atelognathus praebasalticus
Atelognathus reverberii
Lyciasalamandra helverseni
Ololygon peixotoi
Pelophylax cerigensis
Pleurodema somuncurense

Supplementary Table 4.

Numbers of species and percentages of species threatened of major reptile groups (top) and tetrapod classes (bottom) that occur in caves/subterranean and marine coastal habitats. Marine coastal species included are those that make use of coastal terrestrial habitats (e.g., marine iguana); species were not included if they inhabit the ocean for a significant part of their life cycle (e.g., marine turtles).

	**Crocodiles**		**Lizards**		**Snakes**		**Turtles**	
**Habitat**	Species	% Threatened	Species	% Threatened	Species	% Threatened	Species	% Threatened
Caves/Subterranean	0	0.0	43	36.8	7	35.0	0	0.0
Marine Coastal	2	50.0	60	24.7	11	21.6	9	60.0

	**Amphibians**		**Birds**		**Mammals**		**Reptiles**	
**Habitat**	Species	% Threatened	Species	% Threatened	Species	% Threatened	Species	% Threatened
Caves/Subterranean	102	51.0	24	12.5	485	18.4	50	36.5
Marine Coastal	29	10.3	215	29.8	92	35.9	82	26.2
