# Supplementary material for: A global reptile assessment highlights shared conservation needs of tetrapods
Source: Nature. 2022 Apr 27;605(7909):285–90. doi: 10.1038/s41586-022-04664-7 (PMC9095493; doi:10.1038/s41586-022-04664-7)
Supplement: Supplementary file 2 — Reporting Summary [file 41586_2022_4664_MOESM2_ESM.pdf]

nature portfolio

Corresponding author(s): Bruce E Young

Last updated by author(s): Jan 31, 2022

Reporting Summary

Nature Portfolio wishes to improve the reproducibility of the work that we publish. This form provides structure for consistency and transparency in reporting. For further information on Nature Portfolio policies, see our Editorial Policies and the Editorial Policy Checklist.

Statistics

For all statistical analyses, confirm that the following items are present in the figure legend, table legend, main text, or Methods section.

n/a	Confirmed	
☒	☐	The exact sample size (*n*) for each experimental group/condition, given as a discrete number and unit of measurement
☒	☐	A statement on whether measurements were taken from distinct samples or whether the same sample was measured repeatedly
☐	☒	The statistical test(s) used AND whether they are one- or two-sided<br>*Only common tests should be described solely by name; describe more complex techniques in the Methods section.*
☒	☐	A description of all covariates tested
☒	☐	A description of any assumptions or corrections, such as tests of normality and adjustment for multiple comparisons
☒	☐	A full description of the statistical parameters including central tendency (e.g. means) or other basic estimates (e.g. regression coefficient) AND variation (e.g. standard deviation) or associated estimates of uncertainty (e.g. confidence intervals)
☐	☒	For null hypothesis testing, the test statistic (e.g. *F*, *t*, *r*) with confidence intervals, effect sizes, degrees of freedom and *P* value noted<br>*Give P values as exact values whenever suitable.*
☒	☐	For Bayesian analysis, information on the choice of priors and Markov chain Monte Carlo settings
☒	☐	For hierarchical and complex designs, identification of the appropriate level for tests and full reporting of outcomes
☒	☐	Estimates of effect sizes (e.g. Cohen's *d*, Pearson's *r*), indicating how they were calculated

Our web collection on statistics for biologists contains articles on many of the points above.

Software and code

Policy information about availability of computer code

Data collection	No code was used for the data collected for this study.
Data analysis	The following open source software and R scripts were used to analyze the data: Zonation (version 4), zonator (version 0.6.0), caper (version 0.5.2). Custom scripts were developed to work with the Zonation software and zonator scripts and to analyze phylogenetic diversity, and are available at https://zenodo.org/record/5974891. Custom python scripts were developed to analyze the spatial distribution data and are permanently available to the public at https://transfer.natureserve.org/download/Publications/Global_Reptiles/. No software other than Excel was used to analyze the tabular data on Red List category, habitats, and threats.

For manuscripts utilizing custom algorithms or software that are central to the research but not yet described in published literature, software must be made available to editors and reviewers. We strongly encourage code deposition in a community repository (e.g. GitHub). See the Nature Portfolio guidelines for submitting code & software for further information.

Data

Policy information about availability of data

All manuscripts must include a data availability statement. This statement should provide the following information, where applicable:

- Accession codes, unique identifiers, or web links for publicly available datasets
- A description of any restrictions on data availability
- For clinical datasets or third party data, please ensure that the statement adheres to our policy

Taxonomic data for reptiles were from the Reptile Database (http://www.reptile-database.org/index.html). All spatial and tabular data for the tetrapod analyses are permanently available to the public at https://transfer.natureserve.org/download/Publications/Global_Reptiles/. Trees used for the PD analyses are available at https://zenodo.org/record/5974891. In addition, assessment data, including range maps, for all tetrapods are publicly available on the IUCN Red List of Threatened

Species website (https://iucnredlist.org). Occasionally, where a species may be threatened from over-collection, sensitive distribution information is not publicly available. Protected areas boundaries were from the World Database of Protected Areas, available at https://www.protectedplanet.net.

Field-specific reporting

Please select the one below that is the best fit for your research. If you are not sure, read the appropriate sections before making your selection.

☐ Life sciences ☐ Behavioural & social sciences ☒ Ecological, evolutionary & environmental sciences

For a reference copy of the document with all sections, see nature.com/documents/nr-reporting-summary-flat.pdf

Ecological, evolutionary & environmental sciences study design

All studies must disclose on these points even when the disclosure is negative.

Study description

This study examined the extinction risk status of reptiles globally. Data were compiled to address the IUCN Red List criteria, including the development of species range maps. The data were analyze geographically to compare patterns of threat status and potential loss of phylogenetic diversity with other tetrapod groups, as well as with software to address questions about surrogacy of threatened birds, mammals, and amphibians with threatened reptiles. Simple 2x2 contingency tables were used to test for association between habitat use and threat status.

Research sample

For this study, a research sample was an IUCN Red List assessment of a species. Assessments included information about the taxonomic hierarchy, distribution (including a GIS range map), habitats used, threats, and Red List criteria used to assign an extinction risk category.

Additional data sets used were:

The Reptile Database (2020 version, a taxonomic listing of known reptile species descriptions)
IUCN Red List of Threatened Species (version 2020-1, for bird, mammal, and amphibian ranges, habitats, and threats)
Squamate phylogenetic tree (Tonini et al. 2016)
Turtle and crocodile phylogenetic tree (Colston et al. 2020)
Mammal phylogenetic tree (Upham et al. 2019)
Bird phylogenetic tree (Hedges et al. 2015)
Amphibian phylogenetic tree (Jetz et al. 2018)
World Database of Protected Areas (a spatial database of the protected areas in each country)

Sampling strategy

No sampling strategy was used -- we examined all known species of reptiles (aside from some that were described after regional assessments took place).

Data collection

Most data collection took place during regional workshops during which facilitators queried field scientists familiar with species for information about species. Information was entered by the facilitator via computer directly into the Red List database (known as the Species Information Service, or SIS) either in an offline version that was later uploaded to the central version (2004-2013) or directly into the online version (2014-2019). In some cases, the Red List Authority for the IUCN Specialist Group covering a specific group of reptiles completed the assessments for the species in their purview, again via computer to the IUCN Red List database. In all cases, the facilitator or data recorder had previously received training in the proper use of the Red List Categories and Criteria.

The data collectors included manuscript authors Bruce Young, Neil Cox, Phil Bowles, Thomas Brooks, Monika Böhm, Mike Hoffmann, Craig Hilton-Taylor, Marcelo Tognelli, Richard Jenkins, Dave Chapple, Carla Eisemberg, Mark Auliya, Tandora Grant, Kate Sanders, and Krystal Tolley. Additional data collectors were: Ana Nieto, Melanie Bilz, Nieves García, Tulia Defex, Jamie Carr, Monni Böhme, Rossana Merizalde, Nicholas Macfarlane, Jing Zhang, Sarah Sutcliffe, S. R. Ganesh, Srinivasulu Chelmala, Janice Chanson, Caroline Pollock, and Naamal De Silva, Jorge Rodriguez, Margaret Ormes, Geoff Hammerson, Matt Foster, Lily Rodriguez, and Penny Langhammer.

Timing and spatial scale

Collection of data for the reptile assessments initiated 13 December 2004 and concluded on 25 October 2019. The spatial scale of the data collection was global, covering all terrestrial and marine areas where reptiles are known to occur (i.e., excluding polar regions). Data collection primarily took place in remote and in-person assessment workshops, which occurred as funding, logistics, and local scientist availability allowed at an average rate of 3 per year (see Supplementary Table 1 for list of assessment events).

Data exclusions

No data were intentionally excluded from the analysis -- assessments of all known species were included. However, because systematists continue to describe new species, some of these recently described species were left out of the analysis.

Reproducibility

Species assessments went through two levels of review post workshop to improve reproducibility and consistency. First, a scientist familiar with the species but not involved in the assessment reviewed the account for biological accuracy and accurate application of the Red List criteria. Once the assessors revised the assessment satisfactorily, staff from the IUCN Red List Unit reviewed the assessment primarily for accurate application of the Red List criteria. The assessors revised the assessment again, if necessary, to satisfy any concerns of the IUCN Red List Unit before the assessment was finalized. These review processes succeeding in verifying the assessment data to the satisfaction of the original assessor(s), reviewer, and IUCN staff.

No attempts were made to reproduce the original assessments themselves.

Randomization

No randomized groups were used in this study. The study covered the entire "population" (i.e., not a sample) of reptile species known at the time that the study took place. For this same reason, there were no relevant covariates to consider in the analyses.

Blinding	Blinding was not relevant to this study, which did not involve experimental analysis.

Did the study involve field work? ☐ Yes ☒ No

Reporting for specific materials, systems and methods

We require information from authors about some types of materials, experimental systems and methods used in many studies. Here, indicate whether each material, system or method listed is relevant to your study. If you are not sure if a list item applies to your research, read the appropriate section before selecting a response.

Materials & experimental systems

n/a	Involved in the study	
☒	☐	Antibodies
☒	☐	Eukaryotic cell lines
☒	☐	Palaeontology and archaeology
☒	☐	Animals and other organisms
☒	☐	Human research participants
☒	☐	Clinical data
☒	☐	Dual use research of concern

Methods

n/a	Involved in the study	
☒	☐	ChIP-seq
☒	☐	Flow cytometry
☒	☐	MRI-based neuroimaging
